# PEEK in Harsh Oil and Gas Environments: Applications and Chemical Aging Response

**DOI:** 10.3390/polym18162013

**Published:** 2026-08-19

**Authors:** Wael Badeghaish, Ahmed Wagih, G. Lubineau

**Affiliations:** 1 Mechanics of Composites for Energy and Mobility Lab, King Abdullah University of Science and Technology (KAUST), Thuwal 23955-6900, Saudi Arabiaahmed.abdelhady.1@kaust.edu.sa (A.W.); 2 Upstream Advanced Research Center, Saudi Aramco, Dhahran 31311, Saudi Arabia; 3 Mechanical Design and Production Department, Faculty of Engineering, Zagazig University, Zagazig P.O. Box 44519, Sharkia, Egypt

**Keywords:** PEEK aging in oil and gas, scCO_2_ aging, diffusion kinetics, mechanical response

## Abstract

The oil and gas (O&G) industry is increasingly adopting non-metallic materials for pipelines and downhole components to mitigate corrosion, reduce maintenance costs, and improve performance in harsh service environments. Among high-performance polymers, polyether-ether-ketone (PEEK) has attracted significant attention owing to its excellent mechanical properties, thermal stability, and chemical resistance, making it a promising candidate for aggressive downhole applications. However, exposure to acids, hydrocarbons, water, CO_2_, and supercritical CO_2_ under high-pressure/high-temperature conditions can alter its microstructure and mechanical performance, necessitating a comprehensive understanding of its long-term behavior. This review summarizes the microstructure, properties, and current applications of PEEK in the O&G industry, including its emerging use in additive manufacturing. It further examines the fundamental mechanisms of gas and liquid diffusion, aging processes (physical, chemical, and thermal), and their effects on the morphology, thermal behavior, and mechanical properties of PEEK. By consolidating findings from studies conducted under representative O&G environments, this review identifies current knowledge gaps and future research priorities, providing guidance for the selection, qualification, and design of PEEK components for demanding oil and gas applications.

## 1. Introduction

In the oil and gas (O&G) sector, corrosion in metallic materials poses a serious threat to plant integrity. Downhole environments are among the most difficult in the business because they are inherently corrosive. Temperature, corrosive gases (such as carbon dioxide (CO_2_) and hydrogen sulfide (H2S), water chemistry, and flow velocity can corrode the interior of pipes [1,2,3]. This issue can be mitigated using the right materials, coatings, corrosion inhibitors, and interior linings for metallic pipes [3].

In recent years, the use of NM polymer-based materials for pipelines has garnered attention in the O&G sector as an innovative means to transfer pressured fluids from onshore surface locations to offshore subsea environments and ultimately to the downhole. A recent economic study reported that the use of NM pipes in various industries is projected to yield significant cost savings over their entire life cycle and increase oil production [4,5]. NM materials have long been employed in a restricted capacity in downhole (subsurface) applications, mostly for activities such as water production and injection, and shallow hydrocarbon wells. Moreover, 3D-printed polymers are expected to replace metallic materials in electrical submersible pumps (ESPs) and sealing for enhancing their efficiency [6].

In the O&G industry, particularly downhole environments, NM materials offer several benefits, including less-frequent maintenance and work-over, long-lasting equipment, and easier installation [4,5,6]. However, these materials face challenges due to underground conditions, such as high pressures and temperatures (HPHTs) and exposure to various fluids and chemicals [3,7]. The use of chemicals and CO_2_ in enhanced oil recovery (EOR) and acids to clean oil wells adds to these complexities, as they can interact with polymers and change their structure. While these polymers have shown promise, they can still fail due to exposure to acid gases and other contaminants. Therefore, it is crucial to understand the interaction of NM materials with harsh underground environments to ensure their reliability and effectiveness for long-term use [3].

**Figure 1:**
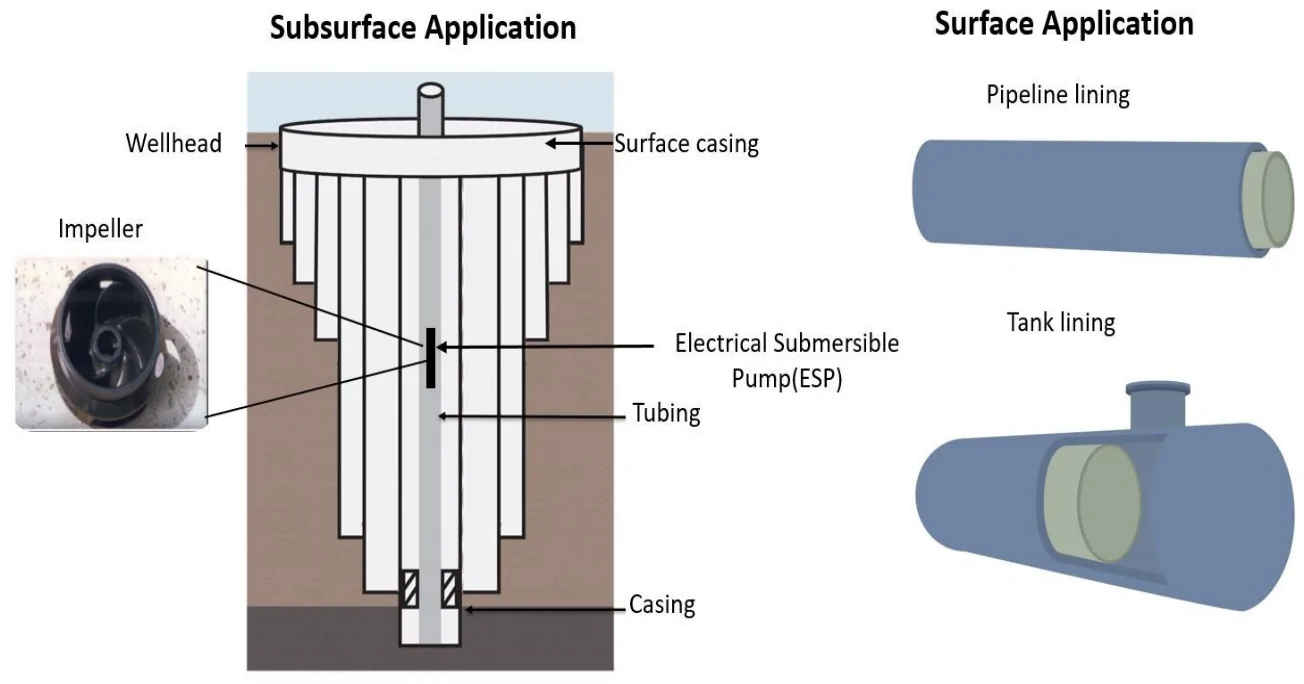
Non-metallic application for subsurface and surface applications.

Figure 1 illustrates representative surface and downhole applications of NM materials in the O&G industry. For example, in surface applications, polymer materials are used as internal liners in carbon steel pipelines or as a pressure-containing layer on full composite flowlines for the transportation of CO_2_, hydrocarbons, and fluids produced under moderate pressure and temperature conditions. In contrast, downhole applications involve much harsher environments, where NM materials are used as liners in gas wells, slicklines, wirelines, drilling jars, and drill pipes [8,9]. These components are exposed to high pressures, high temperatures, CO_2_ in supercritical state, acidic fluids, and other aggressive environments. The combined effects of these service conditions can promote the diffusion of gases and liquids, leading to gradual microstructural changes that may influence the long-term microstructure and mechanical performance of polymer materials [10,11,12,13]. Therefore, selecting materials with high thermal stability, chemical resistance, and low fluid uptake is essential.

Among engineering thermoplastics, high-performance polymers such as PEEK have demonstrated excellent performance under demanding downhole conditions [8], making them a valuable material for O&G applications. PEEK offers excellent thermal stability, chemical resistance, and relatively low gas uptake compared with many engineering thermoplastics. Although PEEK is more expensive than conventional polymers, it is widely used in critical applications because its superior durability and long service life can outweigh the higher initial material cost. Nevertheless, the microstructure and mechanical performance of PEEK can still be influenced by prolonged exposure to high temperature, high pressure, aggressive fluids, and organic solvents. Depending on the service conditions and exposure time, PEEK can undergo swelling, secondary crystallization, and even cracking [11,12]. Therefore, a comprehensive understanding of its behavior under these coupled service environments must be further elucidated [10,11].

**Figure 2:**
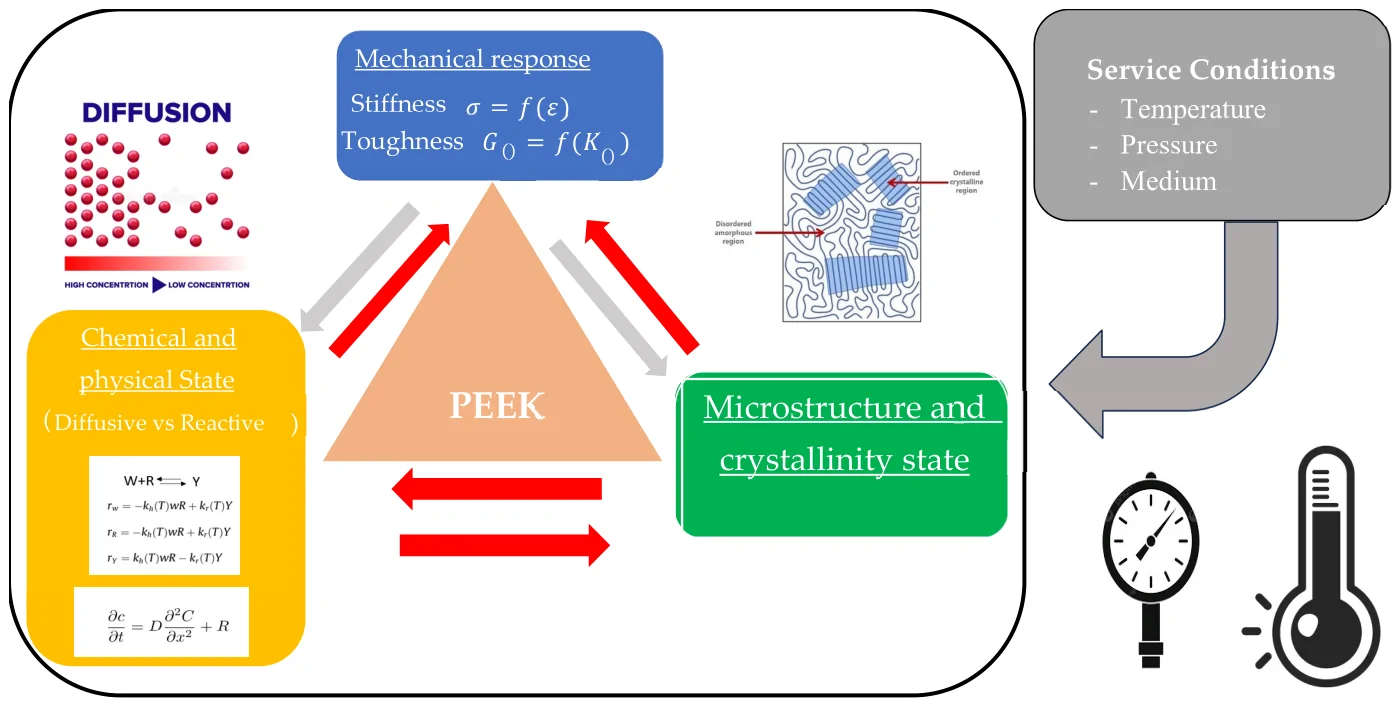
Multi-physics effects on PEEK aging exposed to scCOC_2_ and acidic conditions. particularly under extreme conditions [9].

This review provides an overview of PEEK applications in the O&G industry under harsh service conditions, including supercritical CO_2_ (scCOC_2_), chemical exposure, and other aging environments. It summarizes current studies on diffusion, aging mechanisms, and their effects on the microstructure and mechanical performance of both extruded and 3D-printed PEEK. As illustrated in Figure 2, this review links service conditions with diffusion, microstructural evolution, and the resulting mechanical performance of PEEK. This review also identifies current knowledge gaps and highlights future research directions for the reliable use of PEEK in demanding O&G applications.

## 2. PEEK Microstructure and Properties: An Overview

PEEK is a high-performance semi-crystalline thermoplastic invented in 1978 [14]. PEEK was commercialized in the 1980s for industrial uses [14]. It has various properties with different degrees of crystallinity, high average molecular weight (20,000–40,000 g/mol), and high mechanical modulus (Young’s modulus of 3.6–4.1 GPa); while PEEK thin films have a Young’s modulus of 2.4 GPa [15]. Moreover, PEEK has a *T_g_* of 143 °C and a crystalline melting temperature in the range of 336–343 °C [15,16]. These characteristics contribute to its excellent thermal stability, mechanical strength, and stiffness, making it ideal and reliable for demanding applications in the O&G industry.

**Figure 3:**
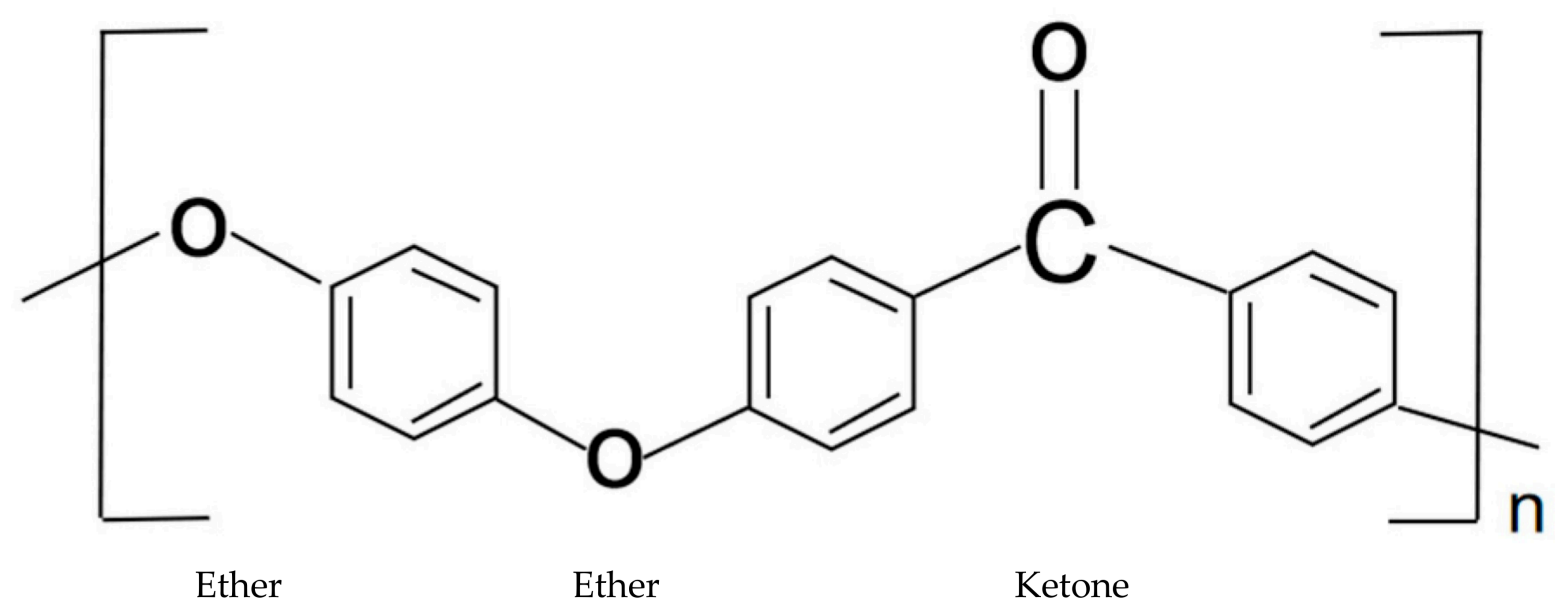
PEEK with repeat unit structure composed of ether and ketone functional groups [20].

The primary monomers used for PEEK synthesis are 4,4′-difluorobenzophenone and hydroquinone. These monomers undergo a nucleophilic aromatic substitution to form long polymer chains containing ether and ketone linkages through a step-growth polymerization process [17,18]. Jin et al. [19] defined the unit cell with a three-ring repeat unit in PEEK with an orthorhombic unit cell structure. PEEK is a linear aromatic polymer, consisting primarily of phenylene rings interconnected by ketone (C=O) and ether (C-O-C) groups [19,20] (Figure 3). The presence of aromatic rings contributes to the rigidity and high thermal stability, while the ether linkages introduce a degree of flexibility. They act as ’hinges’ that allow rotational freedom, which contributes to the toughness of the polymer. The ketone groups enhance intermolecular interactions through dipole–dipole forces, contributing to the high mechanical strength of PEEK [19]. These structural characteristics largely determine the behavior of PEEK under harsh environments, including exposure to scCO_2_ and acidic media.

**Figure 4:**
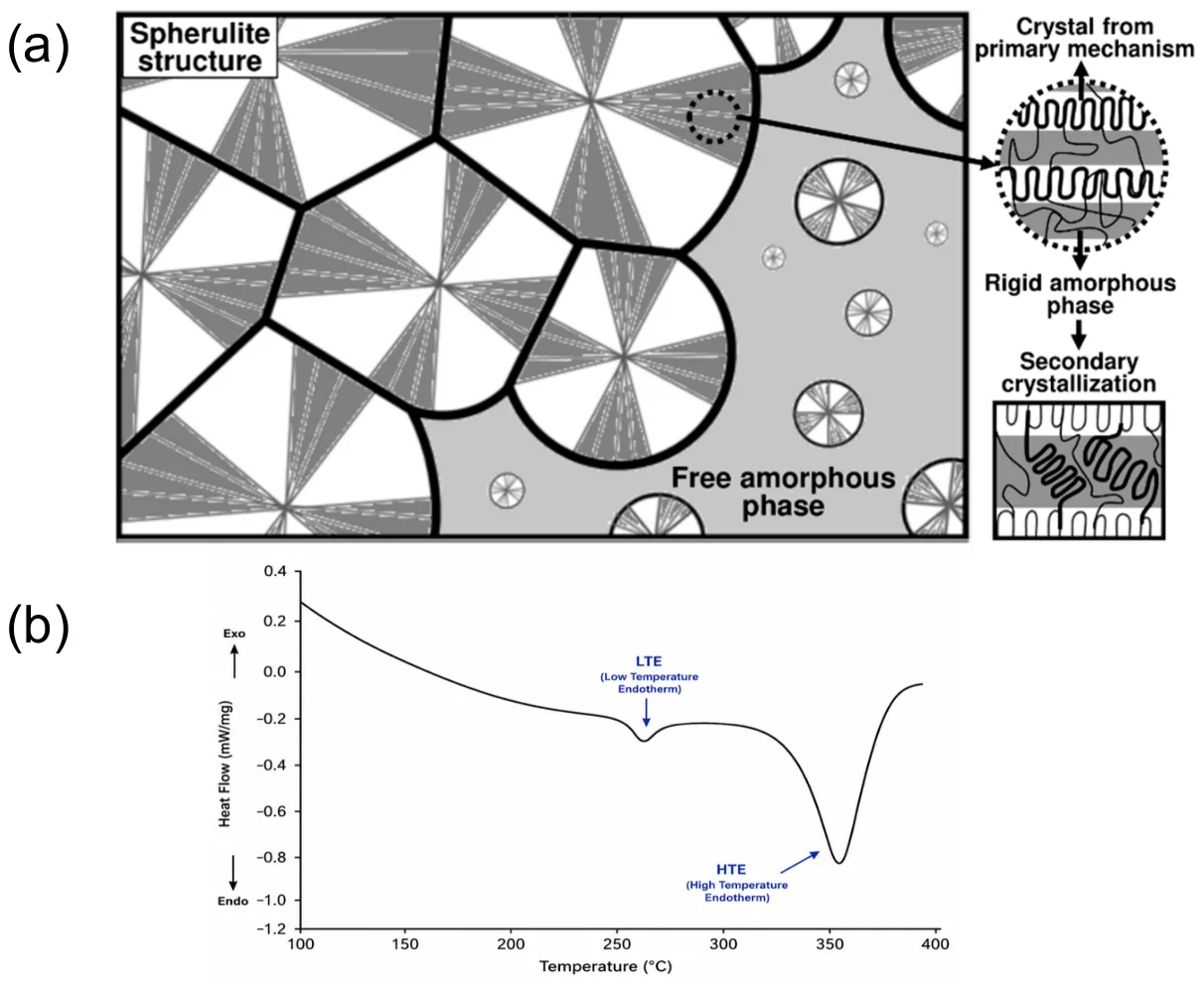
PEEK crystallization: (**a**) schematic representation of the primary and secondary crystallization mechanisms in PEEK [21], and (**b**) double melting behavior of PEEK observed in DSC.

The crystallization behavior of PEEK is influenced by processing conditions such as temperature, cooling rate, and molecular structure. Primary crystallization forms the main crystalline phase, whereas secondary crystallization occurs later within the amorphous regions between lamellae. PEEK crystallizes in two stages, as illustrated in Figure 4a [21]. During primary crystallization, crystal nuclei form and grow into spherulites composed of crystalline lamellae and amorphous regions. Once these spherulites meet, secondary crystallization continues within the remaining amorphous regions, leading to thicker lamellae and a slight increase in the overall crystallinity of PEEK [21]. During thermal analysis, secondary crystallization is often identified by the appearance of a low-temperature endotherm (LTE), while the main crystalline phase corresponds to the high-temperature endotherm (HTE) [22]. Both peaks contribute to the overall crystallinity of PEEK, as illustrated in Figure 4b. As crystallization proceeds, spherulites grow and create additional interlamellar regions where secondary crystallization can occur. Higher crystallization temperatures generally promote larger spherulites and a greater degree of secondary crystallization [22]. The growth of spherulites is governed by general polymeric nucleation theory. At lower temperatures, the formation of new spherulite nuclei is favored over the growth of existing spherulites, leading to a high number of small spherulites [21,22,23,24]. In contrast, at higher temperatures, spherulitic growth predominates over the formation of new nuclei, resulting in fewer but larger spherulites, as illustrated in Figure 4a [21].

## 3. PEEK and O&G Operations

This section aims to illustrate the harsh conditions and challenges encountered in the O&G industry by providing real-world examples of oil field conditions, such as CCS and acid injection for well cleaning, which may pose challenges when interacting with polymers like PEEK. Moreover, it discusses specific PEEK structures used in oil applications, including liners, full tubing, and 3D-printed components.

### 3.1. O&G Operational Conditions

In oil and gas (O&G) operations, both surface and downhole environments pose significant challenges due to extreme pressures, high temperatures, and chemically aggressive substances such as hydrogen sulfide, carbon dioxide, brine, and hydrocarbons. These conditions, particularly in HPHT gas wells, can reach up to 150 °C and 7500 psi at depths around 7572 m in some Middle East fields [25]. Such severe environments demand materials with exceptional resistance and durability to ensure reliable performance in applications like CO_2_ transport and storage.

A major challenge in the O&G industry is that HCl used for acid washing and matrix acidizing can interact with liners and downhole tubulars, potentially altering their microstructure. In general, HCl is used to clean tubulars and wellbores and to improve productivity by removing formation damage near the wellbore. This treatment dissolves deposits, improves fluid flow, and enhances well performance and longevity [26]. In offshore applications, HCl is also used to restore injectivity in produced water reinjection (PWRI) systems. In PWRI, produced water is injected back into the reservoir to maintain pressure and improve oil recovery. Over time, injectivity can decrease because of the buildup of inorganic and organic deposits and microbial activity that block the pore pathways. Acids such as HCl, sulfamic acid, and nitric acid dissolve these deposits, restore permeability, and improve well injectivity [27]. For matrix acidizing near the wellbore, a multi-stage treatment using 20 wt.% HCl and surfactant-based acids was successfully applied in a highly inclined water disposal well. The treatment reduced the skin factor from +22 to nearly zero, increased the injectivity index from 19.7 to 270.2 bbls/psi per day, maintained injectivity for 9 months, lowered the injection pressure by 35%, and reduced the coiled tubing (CT) surface weight by 12.5%, enabling deeper acid placement in extended-reach wells [26].

**Figure 5:**
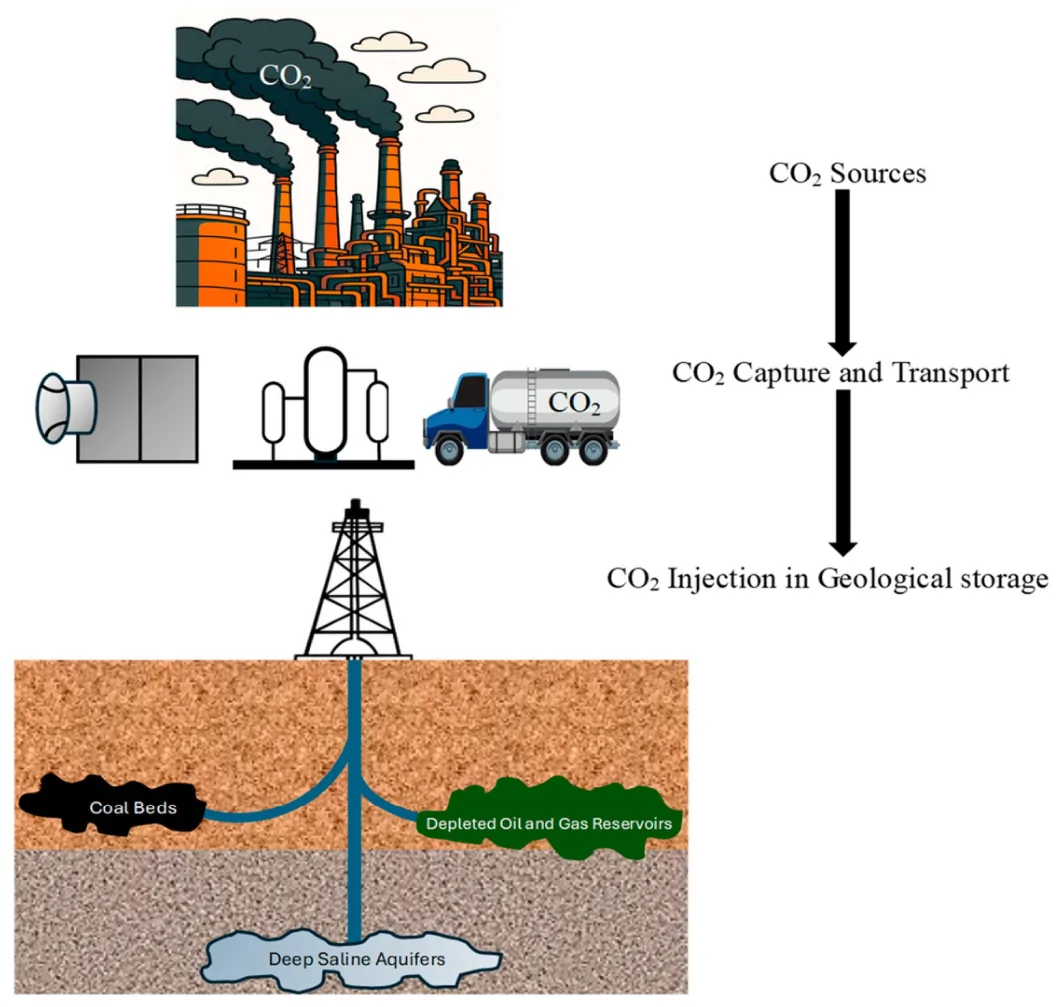
Diagram illustrating an example of the CCS process [28].

Another significant challenge facing sealing and well integrity in the O&G industry is the implementation of Carbon Capture and Storage (CCS) technology, which plays a vital role in reducing emissions from large-scale fossil fuel use. CCS involves capturing CO_2_ generated by energy-related and industrial activities, treating it to remove impurities, and injecting it into a designated storage site for long-term isolation from the atmosphere. This process is illustrated in Figure 5 [28], highlighting the broad scope of CCS research and its role in addressing environmental challenges.

Each stage of the CCS process involves distinct phases and operating conditions [29]. During the CCS process, CO_2_ is first transported through pipelines before being injected into the reservoir for carbon storage or EOR. During transportation, CO_2_ is usually maintained in a dense-phase state to reduce the energy required for compression and minimize the risk of phase changes that could lead to complications in the pipeline system [29].

**Figure 6:**
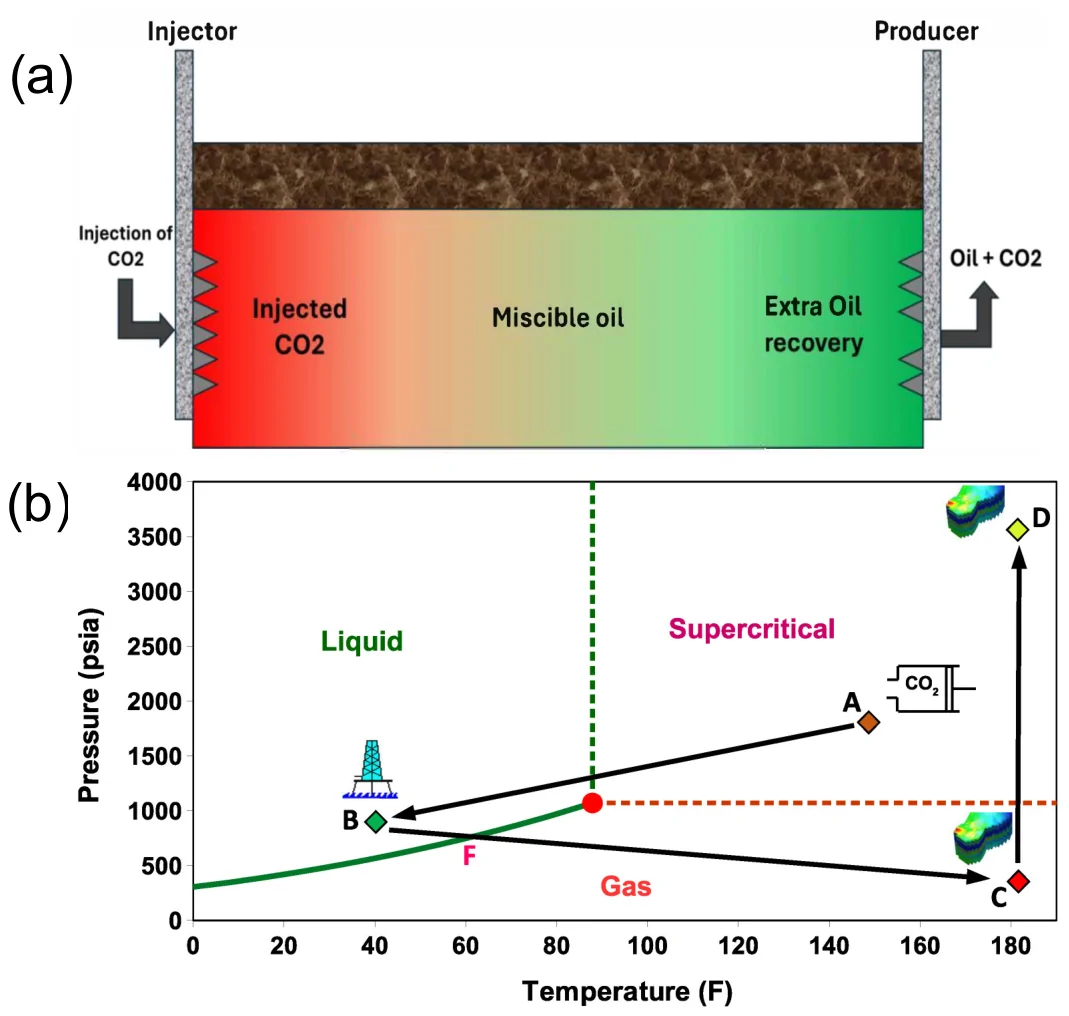
Diagram showing scCO_2_ for EOR: (**a**) schematic of continuous CO_2_ flooding for EOR, highlighting the early breakthrough of injected gas [32,33], and (**b**) P-T diagram of case study of CO_2_ injection [35].

Under these conditions, polymer pipeline liners are mainly affected by CO_2_ permeation, slight swelling, and pressure changes. If the pressure is released rapidly, the absorbed CO_2_ may expand within the polymer, increasing the risk of rapid gas decompression (RGD), particularly in sealing applications [3,30]. After transportation, CO_2_ is injected through downhole tubulars into deep geological formations, where the higher pressure and temperature maintain it in the supercritical state [29]. Under these conditions, scCO_2_ can diffuse more readily into a polymer such as PEEK, leading to greater CO_2_ uptake and changes in the polymer structure, such as secondary crystallization and changes in free volume, which may affect its long-term mechanical performance [30]. The supercritical phase is preferred for injection due to its higher density and better solubility in oil, which enhance the EOR process. These properties allow supercritical CO_2_ to penetrate the reservoir more effectively, reduce oil viscosity, and improve overall extraction efficiency [29,31]. Figure 6a illustrates the process of utilizing carbon dioxide and water injection to recover residual oil trapped in a subsurface rock formation for EOR by flushing the oil between wells [32,33]. It illustrates the CO_2_-EOR process, where the red region represents the injected CO_2_. As CO_2_ contacts the reservoir oil, light oil components vaporize into the CO_2_ while CO_2_ condenses into the oil, forming the miscible transition zone shown in the middle. This miscibility improves oil mobilization and displacement, resulting in enhanced oil recovery, represented by the green region [32]. The figure shows how CO_2_ injection can improve fossil fuel recovery while also storing CO_2_ underground [33].

Figure 6b presents representative pressure and temperature conditions for CO_2_ injection, showing that the operating conditions are typically above the critical temperature (31.1 °C) and critical pressure (7.38 MPa), where CO_2_ exists in the supercritical state. These conditions enhance CO_2_ diffusion into polymers and can influence their long-term aging behavior [34,35]. Understanding CO_2_ phase behavior, along with the impact of its composition and impurities, is crucial for identifying potential risks such as corrosion and other forms of material degradation.

### 3.2. PEEK in O&G Applications

Polymer materials have found extensive use in the O&G industry, particularly in composite tubular designs such as Reinforced Thermoplastic Pipes (RTPs) and Thermoplastic Composite Pipes (TCPs). The choice of thermoplastic depends on its ability to retain its properties during long-term exposure to the intended operating environment, rather than on its initial cost. Table 1 summarizes the wide variety of engineering thermoplastics commonly used in O&G applications and compares the key properties that govern their performance in demanding service conditions [30]. There are several key properties that need to be considered for thermoplastic selection, including the *T_g_*, the maximum service temperature, the resistance to aggressive chemicals, the compatibility with high-pressure CO_2_, and the resistance to fluid uptake and swelling.

**Figure 7:**
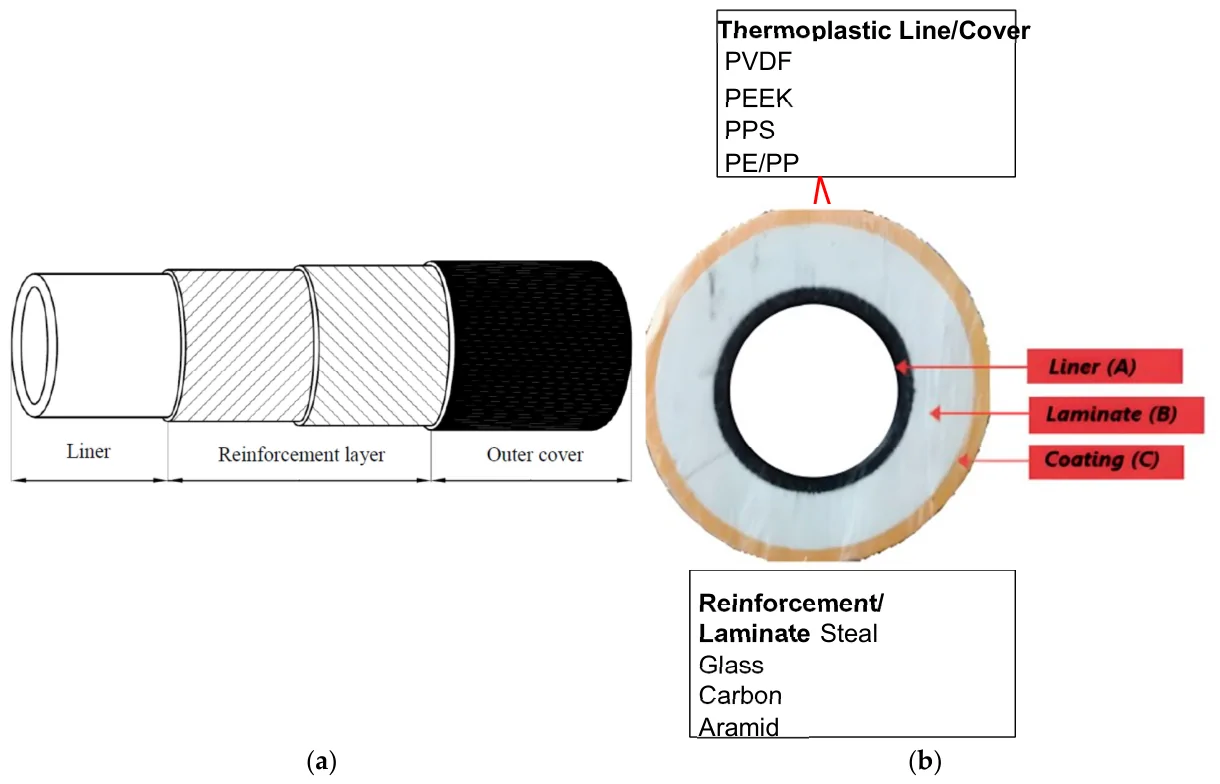
Schematic structure of (**a**) RTPs showing liner, reinforcement, and cover [36], and (**b**) Cross-section showing typical material choices for each RTP layer [37].

RTPs used in onshore and offshore fields typically feature a multi-layer structure consisting of an internal thermoplastic liner, a reinforcement layer, and an outer thermoplastic layer. The typical structure of an RTP is depicted in Figure 7a [36]. Each layer of the RTP consists of a distinct material, carefully selected to meet specific requirements, as illustrated in the cross-sectional view of the RTP layers in Figure 7b [37].

For the liner, various grades of HDPE, suitable for product temperatures up to 65 °C, are commonly used [36]. For higher operating temperatures, RTPs can be designed using alternative thermoplastic materials such as cross-linked polyethylene (PEX), PA, PVDF, and PEEK. The reinforcement layer is usually composed of continuous fibers or tapes and steel wires or tapes, with materials including polyester fiber, glass fiber, carbon fiber, and aramid fiber. The outer protective layer is typically made of HDPE [36,37,38]. These materials are increasingly recognized as suitable and cost-effective solutions for a variety of onshore and offshore applications. The flexible deployment and installation of RTP in onshore applications is reported in the literature [38]. While traditionally more common in downstream operations, the adoption of these composite materials is now expanding into upstream applications by utilizing a diverse range of thermoplastic materials.

For RTP liner applications, material selection is strongly influenced by CO_2_ diffusion and permeability. Compared with other engineering thermoplastics, PEEK generally exhibits lower CO_2_ diffusivity and permeability because of its rigid aromatic backbone, high crystallinity, and low free volume, which restrict gas transport. In contrast, polymers such as HDPE, PA11/12, and PVDF show higher CO_2_ uptake and faster diffusion due to their greater chain mobility and larger amorphous regions, as depicted in Table 1. For all these thermoplastic materials, CO_2_ diffusivity generally increases with temperature as molecular mobility within the amorphous phase becomes more active. Besides its lower diffusion rate, PEEK remains an attractive material for HPHT applications because of its excellent thermal stability, chemical resistance, and mechanical performance under harsh service conditions [3,30,39].

**Figure 8:**
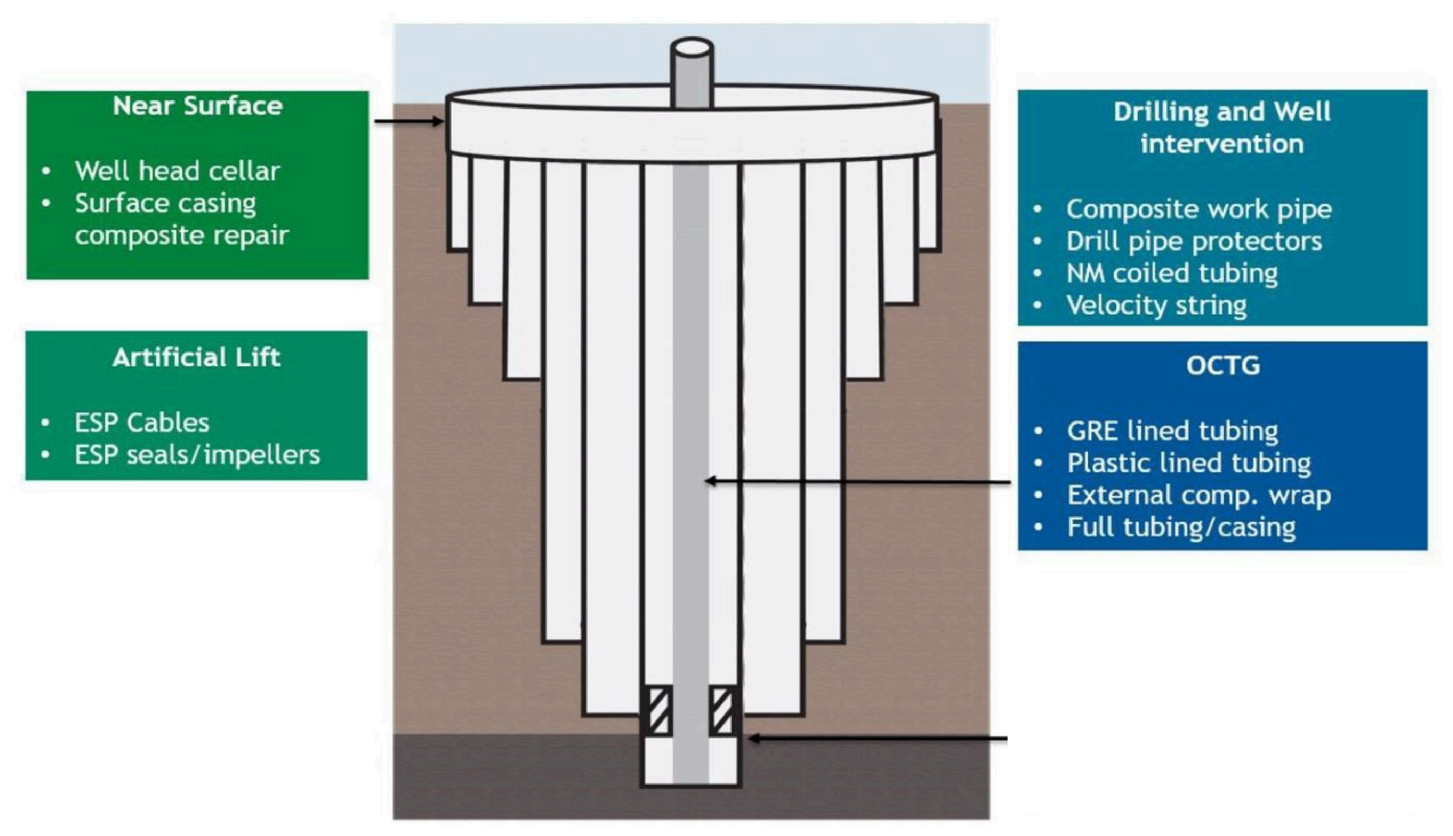
Roadmap for potential application of PEEK in downhole environments [6].

Beyond RTP liners, PEEK has potential for a wide range of downhole applications, as illustrated in Figure 8 [6]. As the industry continues to move towards PEEK with demeaning application due to business need and extended life cycle cost. Several subsurface applications, such as liners for gas wells, well intervention tools, and impellers for ESPs, have already been successfully replaced with PEEK in industry. However, there are additional applications anticipated to be replaced with PEEK in the near future [6]. Despite these advantages, the widespread adoption of PEEK is still limited by its relatively high material cost. Therefore, its use is generally restricted to applications where its superior performance justifies the additional cost. In the O&G industry, PEEK is utilized as a liner for TCP and RTP in short-distance surface applications, where it provides superior long-term performance in aggressive conditions that conventional thermoplastics cannot withstand. In downhole applications, such as in HPHT gas wells, PEEK liners can protect carbon steel tubulars from internal corrosion. This can significantly reduce the need for tubing replacement and workover operations, thereby extending the service life of the tubular. However, the recent advancements in additive manufacturing have made it possible to produce customized PEEK components that are well-suited for high-value, low-volume consumption of PEEK. This is enhancing the supply chain operation. In general, the O&G industry is increasingly turning to PEEK for critical applications where a long lifespan and reduced operating costs are essential.

PEEK can be manufactured in various forms utilizing different processes, including extrusion/plutrusion, which is commonly used for pipelines and spoolable composite tubes. In addition, 3D printing technology can be used to produce complex geometrical items, such as seals and other customized components for drilling and completion applications, offering enhanced design flexibility and precision.

#### 3.2.1. Pultruded PEEK

Pultruded PEEK has become a highly valued material in the O&G industry, especially in upstream operations such as production and drilling, due to its exceptional mechanical strength, thermal stability, and chemical resistance. Pultruded PEEK is increasingly used as a liner material in metallic pipelines and downhole tubulars to provide a critical barrier against aggressive fluids like hydrocarbons, acids, and other corrosive substances in oil and gas extraction [40].

**Figure 9:**
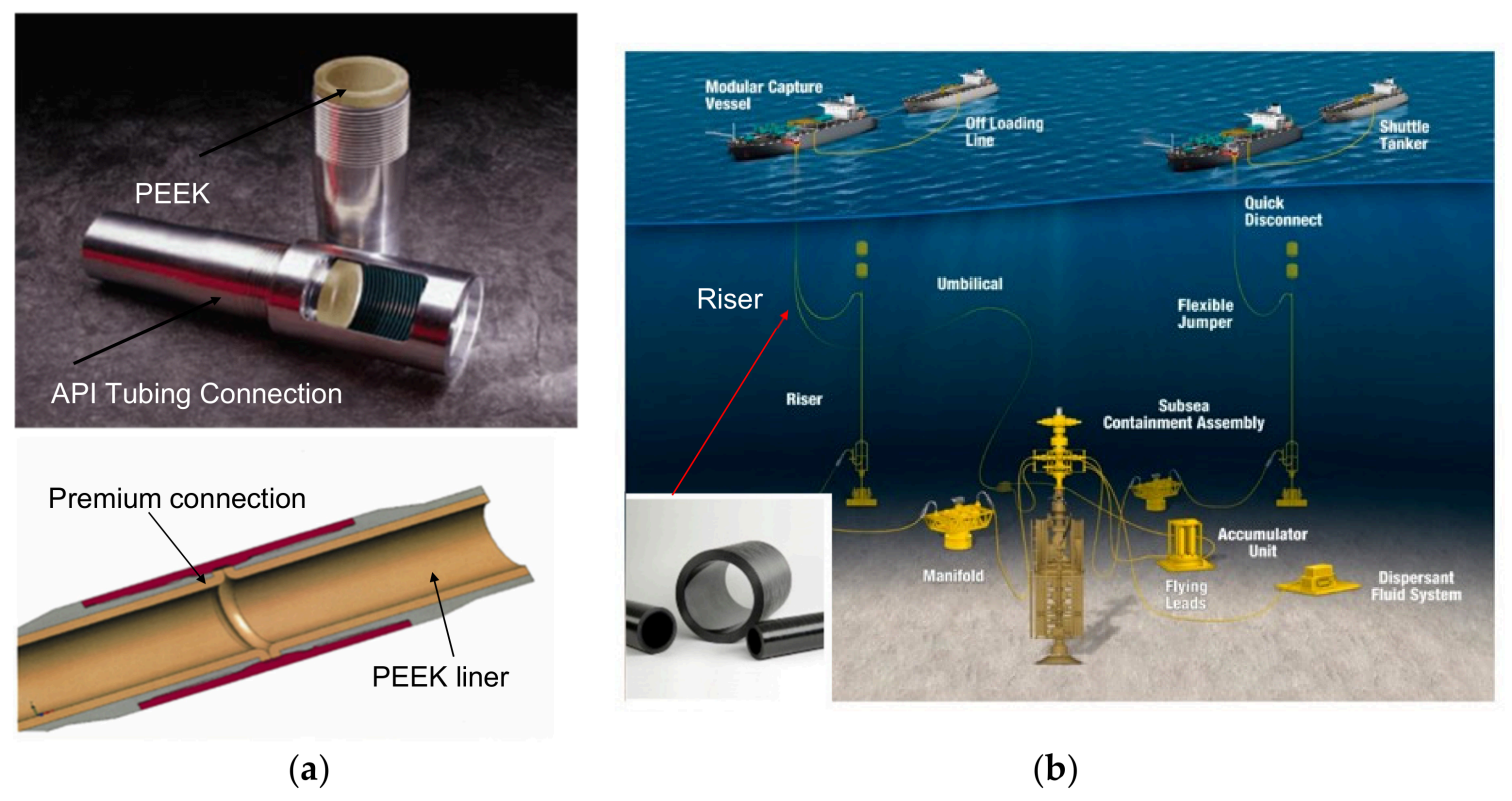
Application of thermoplastics in oil & gas industry: (**a**) Internal thermoplastic liner of carbon steel tubular [40], and (**b**) Composite riser configuration using Magma’s m-pipe solutions for a lightweight and cost-effective riser [44].

Its ability to maintain structural integrity under high temperatures and pressures makes it ideal for HPHT environments where traditional materials may fail. While polyolefins are commonly used for liners in environments up to 99 °C, more demanding conditions require engineering thermoplastics, like PPS, capable of withstanding temperatures up to 175 °C. For the most extreme production environments, reaching up to 260 °C, pultruded PEEK liners were employed [40,41]. Figure 9a illustrates a carbon steel tubular structure lined with a thermoplastic PEEK liner [40]. Thermoplastic-lined material is compatible with both API and premium threaded connections, enabling their use in a wide range of downhole tubular and completion systems. It provides an effective barrier against corrosion, rod-on-tubing wear, and abrasion in harsh production environments. These lined tubulars are widely deployed in beam pump, progressive cavity pump, submersible pump, gas lift, and water injection applications. Their smooth internal surface improves flow efficiency while extending tubing service life and reducing workover frequency [40].

Another application of PEEK reinforced with carbon fiber is TCP. For example, m-pipe (TCP) has been used as a rapid wellhead connection to the well production skid, replacing rigid steel pipes [42]. The industry operator commissioned two m-pipe flexible wellhead jumpers to accelerate installation and allow movement between the well and production skid. Unlike non-bonded flexible pipes, m-pipe resists sour hydrocarbons and gas, making it ideal for onshore production applications. However, m-pipes were also utilized for risers in offshore applications. In general, a composite riser made of PEEK reinforced with carbon fiber (PEEK/CF) has particularly replaced conventional metallic risers in offshore drilling and production to transport fluids (such as oil, gas, or water) between the seafloor and the surface facility (e.g., a drilling rig, platform, or floating production unit). These risers are essential equipment in deep-water and ultra-deep-water operations [43]. For instance, in 2014, pioneering projects in Brazil’s pre-salt offshore regions utilized 3-inch bore fully composite pipes at depths of over 2100 m (6890 feet), which subsequently led to a broader adoption of TCPs in deep-water applications, as shown in Figure 9b [44]. This composite riser (PEEK/CF) replaced metallic ones in offshore applications, offering benefits such as reduced weight, enhanced corrosion resistance, and improved durability [43,44]. Moreover, in sealing applications, PEEK’s excellent resistance to chemical attack and creep under load ensures long-lasting performance even under the harshest conditions and reduces the risk of leaks and equipment failure. The material’s low friction coefficient also contributes to its effectiveness in dynamic sealing applications, where it helps to minimize wear and prolong the service life of components.

**Figure 10:**
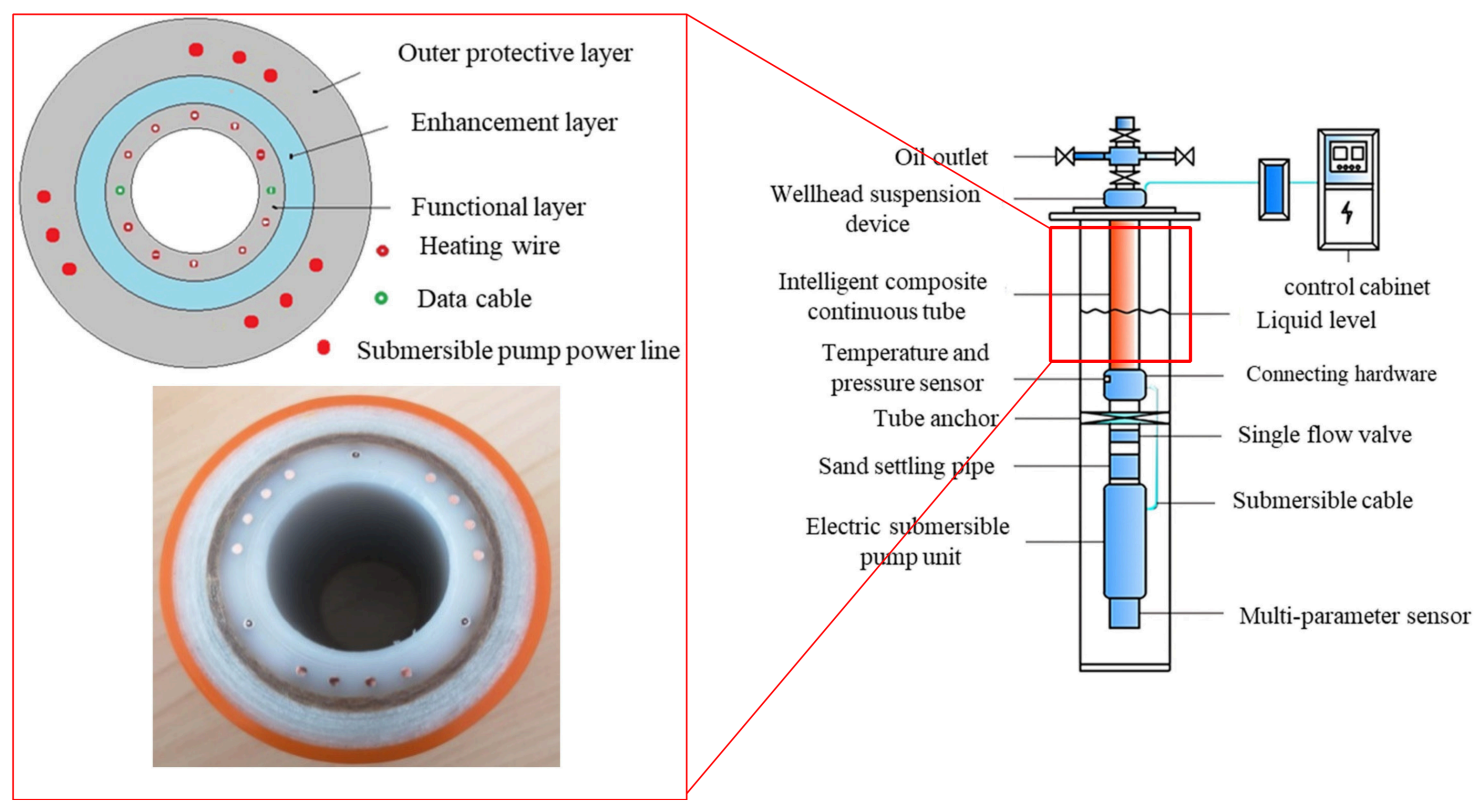
Schismatic diagram of Intelligent composite continuous Pipeline (ICCP) connected with ESP system with cross-section of intelligent nonmetallic cable embedded tubing [45].

In downhole applications, the use of optical fiber sensing techniques for nonmetallic materials in the O&G industry has shown promising results in terms of enhancing operational efficiency and infrastructure monitoring. Intelligent nonmetallic composite continuous pipelines (ICCP) represent a technological advancement for drilling, well intervention, velocity strings, production tubing, and transportation in onshore and offshore oilfields. These flexible spoolable pipelines can incorporate power lines, optical fibers, and other cables to enhance advanced monitoring capabilities. As shown in Figure 10, ICCP with an incorporated inner liner made of PEEK or other polymers connected with ESP can provide real-time data on downhole tools and equipment while protecting the cables during operations. For surface pipelines, this technology offers a cost-effective method for detecting leaks over long distances, which can reduce labor costs and improve operational efficiency [45]. Another innovative use for composites is in the tooling used to measure well performance, namely wireline. This composite wireline is replacing its metallic counterpart to resist corrosion and extend its life cycle cost-effectively. Wirelines are essential tools in subsurface wells, used for logging, maintenance, and evaluation of well integrity. A 6000 m semi-stiff, spoolable composite rod has been developed. This rod can be deployed in highly deviated and horizontal oil, gas, and water injection wells to assess well and reservoir performance. It incorporates multiple fiber optic lines for data transmission and sensing within the wellbore and operates effectively at temperatures up to 150 °C [46].

**Figure 11:**
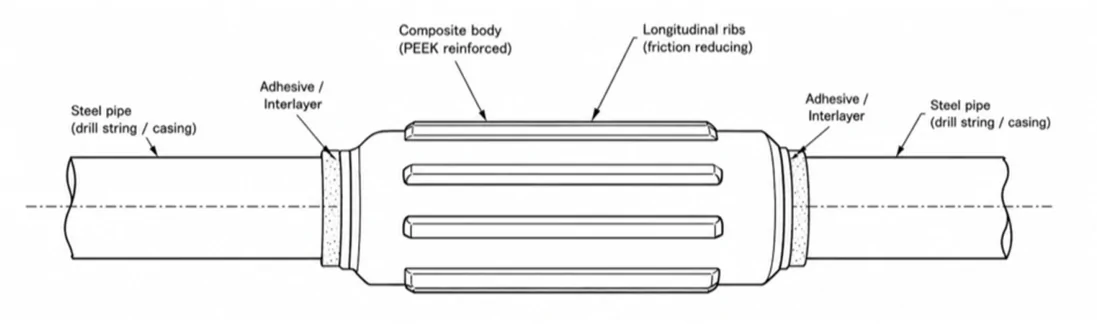
Designed composite centralizers for specific drilling and completion applications.

In deepwater drilling operations, reducing friction and drag is crucial to ensure efficient and safe drilling. One of the key components that can help achieve this is the casing centralizer. Casing centralizers play a crucial role in drilling and well completion operations, helping to reduce torque and drag and improve wellbore hydraulics. Traditional steel centralizers have limitations that can hinder well performance, particularly in challenging drilling environments. To overcome these limitations, composite materials such as PEEK-reinforced or other high-performance thermoplastic materials are being used to make new centralizers, as illustrated in Figure 11. These composite centralizers reduce friction by 44% compared to steel, making them a more efficient option for drilling operations. By using composite centralizers made from advanced materials like PEEK, operators can save costs, improve their overall drilling and completion operations, and achieve better well performance [47].

#### 3.2.2. 3D Printed PEEK

**Figure 12:**
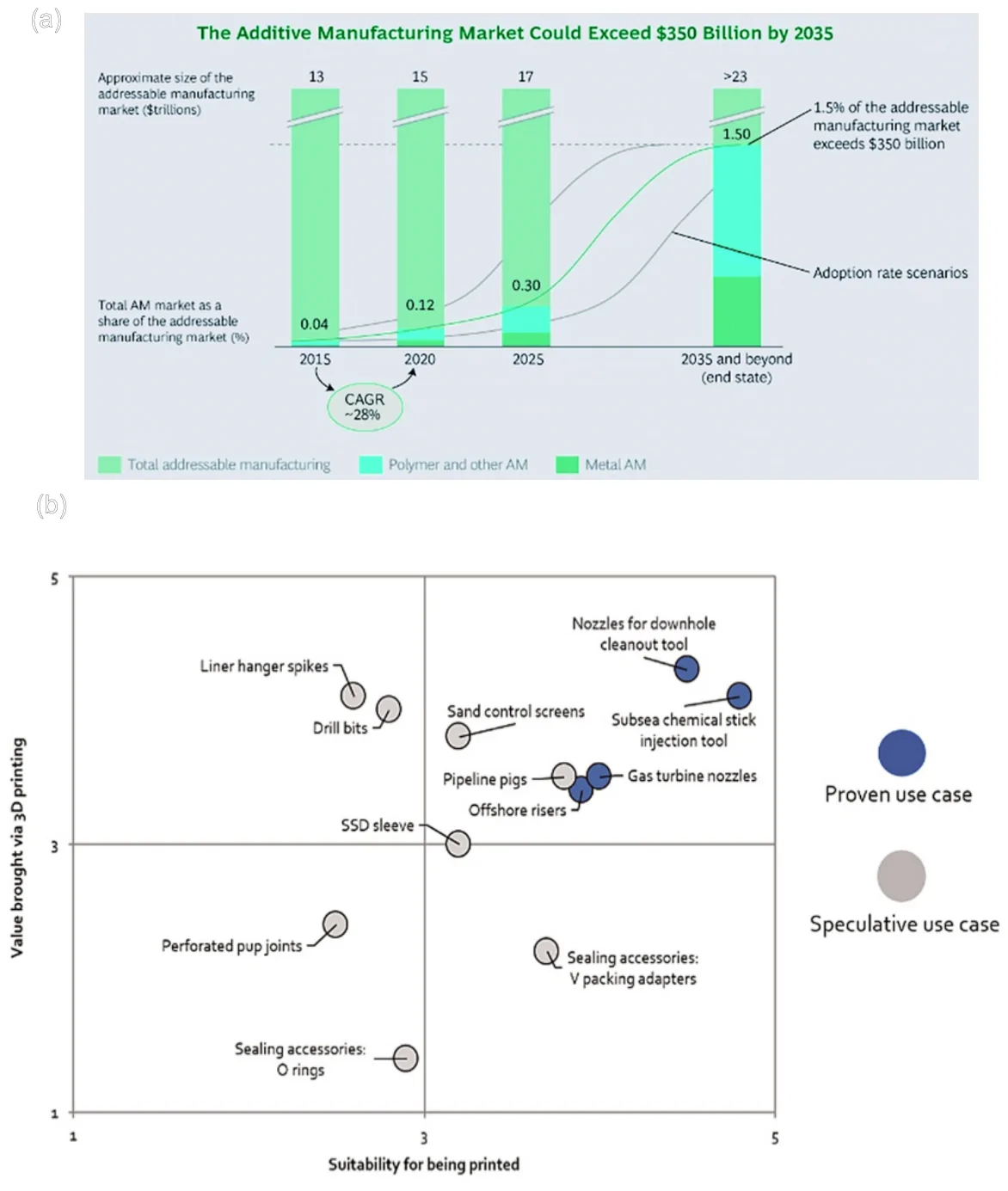
AM market analysis presented by (**a**) Boston Consulting Group, spanning the entire AM value chain, presents data for the middle adoption scenario, and (**b**) Oil and gas applications of AM components [48].

3D printing can considerably impact supply chain management in the O&G industry, rather than manufacturing cheaper machinery or improving their efficiency. The long-term use of O&G equipment in highly remote places causes breakage, and the equipment becomes outdated. The performance of this equipment cannot be assessed in time because they are large in number and do not last as long as the main equipment. 3D printing can help solve this problem. It allows the fabrication of rare or outdated parts as required and thereby facilitates the functioning of equipment [48]. Boston Consulting Group estimated that the 3D printing market was worth around $5 billion in 2015 and would grow rapidly at a rate of nearly 30% each year until 2020. If 3D printing were to be used for about 1.5% of all manufacturing by 2035, its market value could surpass $350 billion, with a significant increase in the amount of additive manufacturing (AM) polymers fabricated compared to the previous year [48]. This can be seen in Figure 12a [48].

The O&G industry can use 3D printing to fabricate twelve final products, as illustrated in Figure 12b [48]. They evaluated these uses based on two main factors: the ease with which the parts could be 3D printed and their benefits. Parts nozzles for oil well cleaning tools, offshore pipe supports (risers), nozzles for gas turbines, and tools for injecting chemicals underwater were successfully fabricated via 3D printing [48].

**Figure 13:**
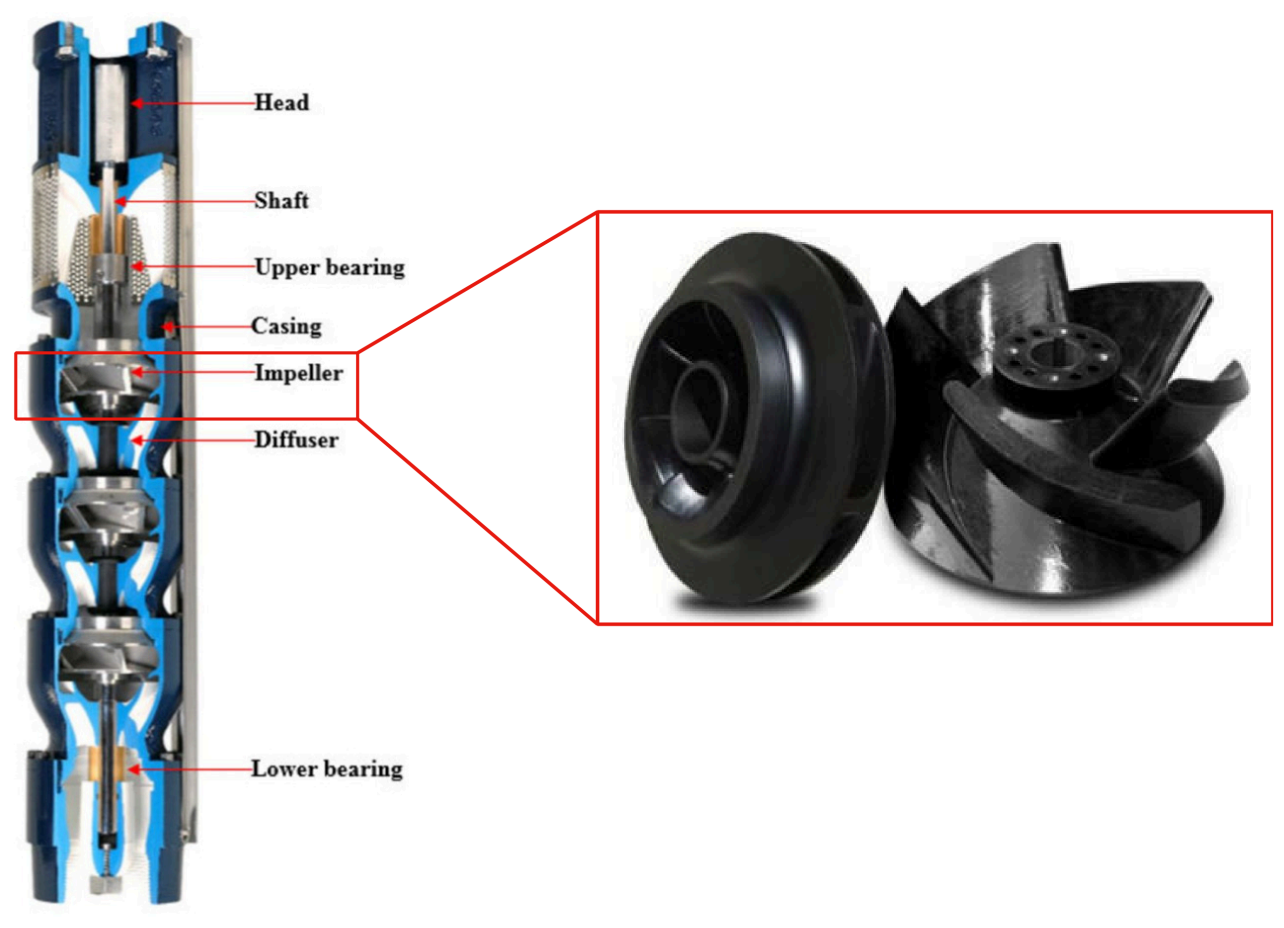
CF-reinforced 3D printed PEEK applications: for upstream ESP impeller [6].

PEEK has been produced by 3D printing, and its ability to withstand extreme weather conditions has been investigated [48,49]. One example of a promising application is the 3D-printed PEEK impeller reinforced with carbon fiber for downhole ESP, which has demonstrated significant benefits by reducing erosion and corrosion issues commonly associated with conventional metallic impellers, as shown in Figure 13. Moreover, another significant application of 3D-printed carbon fiber-reinforced PEEK in the downstream sector is in vertical pump bushings. These bushings are crucial for the reliability of vertical pumps, providing support and minimizing wear; however, they deteriorate over time, leading to maintenance challenges. Stocking all variants of spare parts is costly for large operations, and traditional procurement often leads to excess inventory [50]. 3D printing offers a flexible solution, allowing on-demand production of bushings in the needed sizes and quantities, which reduces waste and storage needs. To demonstrate this, two bushings were 3D printed using Filament Deposition Modeling (FDM) with carbon fiber-reinforced PEEK and could replace the traditional alloy material [50].

**Figure 14:**
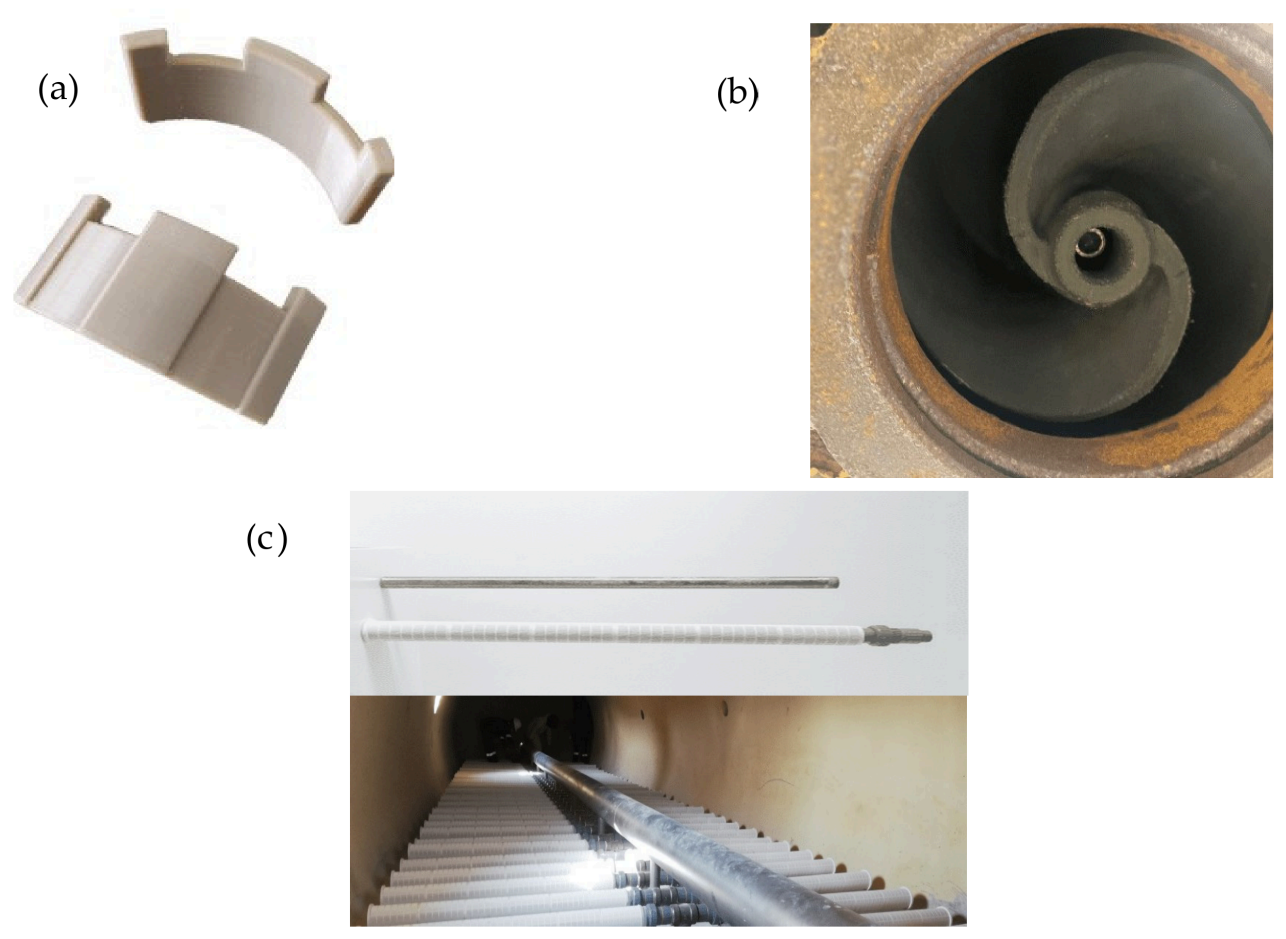
Examples of 3D-printed PEEK parts: (**a**) C-block for ESP motors, (**b**) CF-reinforced polymer for water treatment pump impellers, and (**c**) polymer sand screen for water filtration system [51].

The 3D printed PEEK has been used to manufacture C-blocks for ESP motors as illustrated in Figure 14a. Conventional production of these components requires dedicated tooling and can be time-consuming. The AM provides a practical alternative by reducing production time and allowing rapid fabrication of customized components for ESP applications [51]. To support the adoption of AM in surface applications, the water treatment pump impeller was manufactured from carbon fiber-reinforced nylon using FDM 3D printing as depicted in Figure 14b. The material was selected for its good corrosion resistance and high strength in wet operating conditions. The new design reduced the impeller weight by 18% while maintaining reliable performance. Another example of the adoption of 3D-printed PEEK in downstream and refinery operations is the production of polymer-based sand screens for water filtration systems. Compared with conventional manufacturing, 3D printing enables the fabrication of complex filtration geometries while reducing production time and simplifying design modifications, as illustrated in Figure 14c. This approach improves filtration performance and provides a cost-effective solution operating in corrosive environments [51].

Although 3D printing offers many advantages, the presence of voids remains a challenge. Numerous studies have been conducted to study the effects of thermal processing and optimize the printing parameters to improve the mechanical properties and crystallinity of 3D-printed PEEK [52,53]. However, studies on the aging behavior of 3D-printed PEEK are scarce. Only a few studies reported the effects of heat and moisture aging on the properties of PEEK [49]. El Magri et al. examined the impact of physical and chemical aging on the characteristics of a 3D-printed composite material composed of PEEK/PEI. The printed samples were exposed to a dry heat environment at 120 °C and a moist heat environment at 70 °C with a relative humidity of 85% for 1000 h. The results indicated that aging did not cause any substantial alterations in *T_g_* or the degree of crystallinity, suggesting high thermal stability. Furthermore, the stiffness and modulus of samples that underwent physical aging decreased by approximately 10–11.5% due to the presence of voids [49].

### 3.3. Potential Challenges of the Application of PEEK in O&G Operations

Thermoplastic materials are susceptible to aging in O&G environments due to the harsh environmental factors such as temperature, pressure, and exposure to various media. This aging process manifests in three primary ways. First, there are visual changes, including stains, cracks, and alterations in color. Second, physical properties are affected, leading to shifts in solubility, swelling, thermal resistance, and permeability to water or gas. Finally, mechanical properties are altered, resulting in variations in tensile strength, bending, shear, impact resistance, and elongation. These cumulative effects of aging can ultimately lead to material failure during service [54]. Despite these advantages, the widespread adoption of PEEK in the O&G industry may face several challenges for long-term applications. A significant challenge for PEEK in the O&G industry is its aging behavior under prolonged exposure to harsh downhole environments. These conditions involve extreme pressures, high temperatures, and chemically reactive substances like hydrogen sulfide (H_2_S), water, acids, and hydrocarbons, which could lead to complex chemical interactions that might compromise PEEK’s long-term performance. In applications such as CO_2_ transport, storage, and EOR, the high reactivity of scCO_2_, along with other chemicals present in the subsurface environment, further degrades PEEK. The diffusion of CO_2_ and acids into PEEK during these operations alters its morphology, thermal stability, and mechanical properties over extended exposure periods [3,30,55]. In addition, acid treatment operations aimed at enhancing well production and increasing injectivity have presented potential challenges with downhole tubular liner [56]. The effects of these aggressive conditions on the nano, micro, and macrostructures of Pultruded PEEK structures are not fully understood, which represents a significant gap in the literature. There have been limited studies on how prolonged thermal exposure of PEEK affects its crystallinity, phase morphology, and the potential for microcracking or other forms of structural degradation.

3D printing has the potential to significantly impact supply chain management in the oil and gas industry, beyond just reducing manufacturing costs or enhancing equipment efficiency [48,57]. However, the integration of 3D-printed PEEK parts into O&G equipment adds another layer of complexity. Achieving consistent material properties across printed parts is challenging due to anisotropy, variations in printing parameters, and the presence of voids, all of which could compromise mechanical performance, especially in safety-critical applications. The impact of aging on 3D-printed PEEK under scCO_2_ at high pressures and temperatures is particularly concerning and requires further exploration.

## 4. Diffusion in Semi-Crystalline Polymer

The diffusion of gases and liquids in polymers is a complex phenomenon that is influenced by the material’s microstructure, particularly the content of amorphous and crystalline regions. A thorough understanding of these diffusion processes is essential, as factors such as pressure, temperature, environmental conditions and size of penetrant significantly impact the permeation of substances into polymer matrices. The fundamental formulas governing gas and liquid diffusion in polymers, including the underlying mechanisms and mathematical formulas that describe these phenomena, are described for each section.

### 4.1. Diffusion of Gases in Semi-Crystalline Polymer

Gas diffusion in polymers is a critical factor that affects polymer behavior in various applications, especially under conditions of elevated pressure and temperature. As gas molecules enter a polymer, they travel through its free volume and lead to potential changes in the microstructure. This can manifest as plasticization and changes in the crystallinity, swelling, and mechanical properties. The diffusion process is heavily influenced by the polymer microstructure, which determines how easily gases can dissolve and move through the material [58]. The microstructure of a polymer plays a major role in both the diffusivity and solubility of gases. In order to simulate this diffusion, free-volume theories like the Vrentas and Duda or Fujita models have been extensively used [59,60,61]. These models explain how small molecules diffuse by moving into temporary voids within the polymer, created by local thermal fluctuations. The models can be used to assess how alterations in temperature and pressure affect the free volume for gas diffusion, thereby impacting overall polymer diffusion behavior. Temperature increases generally expand these voids by increasing kinetic energy and chain mobility, thereby facilitating the diffusion process [58]. The effect of pressure on diffusion can differ; it may increase, decrease, or have no impact at all [62]. In semi-crystalline polymers, while the amorphous regions facilitate diffusion, the crystalline regions complicate the molecular path, increasing the tortuosity and significantly affecting diffusion behavior [58,63].

The diffusion of gases in polymers is often modeled using Fick’s second law as an equilibrium equation and Henry’s law as a boundary condition used to determine the solubility of gases under different partial pressures. Fick’s second law was derived from Fick’s first law, which relates to steady-state diffusion (when the concentration does not change with time), where the diffusion flux **J** is proportional to the concentration gradient ∇*c* as follows:

(1) J=−D∇c

where *D* is the diffusion coefficient.

Fick’s second law describes the change in gas concentration over time and space within a polymer. It is represented by the diffusion equation, where the diffusion flux **J** provides the foundational expression for the first form of Fick’s second law.


(2) $$\frac{\partial c}{\partial t}=-\nabla .\;J$$

Substituting **J** from 1 into Fick’s second law 2, the formula can be written in concentration gradient form, as follows:

(3) $$\frac{\partial c}{\partial t}={D\;\nabla }^{2}\;c$$

where $\frac{\partial c}{\partial t}$ is the rate of change in concentration over time, *c* represents the concentration of the diffusing species, *t* denotes time, and ∇^2^*c* is the Laplacian (second spatial derivative) of the concentration. In one-dimensional scenarios, this equation simplifies to:


(4) $$\frac{\partial c}{\partial t}=D\;\frac{\partial^{2}c}{\partial x^{2}}$$

**Figure 15:**
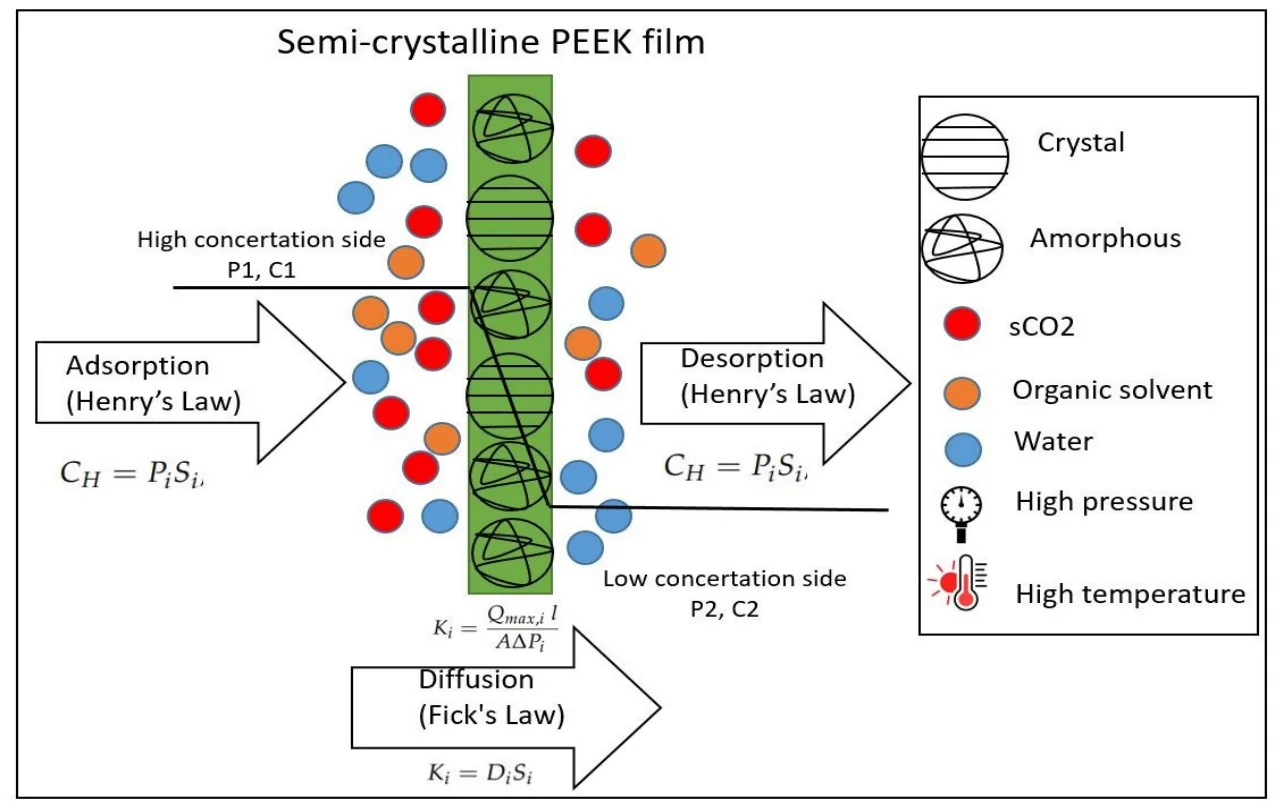
Transport of gas and liquid molecules through a membrane with thickness l by adsorption, diffusion, and desorption.

Typically, diffusion happens through the less orderly, amorphous regions of the polymer, following Fick’s laws, which state that molecules move from high to low concentration areas, as explained in Figure 15.

The chemical affinity between the diffusing substance and the polymer plays a crucial role in gas diffusion. This affinity is often influenced by the similarity in polarity and functional groups between the polymer and the penetrating substance, which directly affects the ease with which the substance diffuses through the polymer. When a substance has a similar polarity or shares functional groups with the polymer, it is more likely to interact with and penetrate the polymer matrix. For instance, a polar solvent or gases would have a higher affinity for a polar polymer, leading to easier diffusion into the polymer [64,65]. Alternatively, permeability measures how easily a substance can pass through a polymer and is dependent on both solubility and diffusivity [58]. The permeability (cm^3^(STP) cm^−1^ s^−1^ bar^−1^) of dense polymers is determined by transient time, which can be expressed in terms of a diffusion coefficient (cm^2^ s^−1^), as well as the affinity of the penetrant for internal surfaces, determined by the solubility coefficient (barg^−1^). Relationships between permeability and solubility in polymer can be described simply as follows:

*K* = *DS* (5)

where *k* is the permeability, and *S* is the solubility coefficient.

Henry’s Law states that the gas concentration at the boundary of a polymer is directly proportional to the partial pressure of the gas, expressed by the equation:

*c*_0_ = *PS* (6)

where *S* is the solubility and *P* is the pressure at the boundary [63]. The Solubility is often measured in units of $\frac{{cm}^{3}\;STP}{{cm}^{3}\;Mpa}$. This unit indicates the volume of gas at standard temperature and pressure (0 °C, 1 atm) (cm^3^STP) dissolved in a cm^3^ of polymer at 1 MPa.

The relationship between activation energy and temperature is a fundamental concept in chemical kinetics and is typically described based on the Arrhenius law [58]. This relation is crucial for understanding the effect of temperature on the rate of chemical reactions and physical processes such as diffusion in materials, including polymers. Molecules must overcome the activation energy barrier to diffuse through a material. According to the Arrhenius relation, higher temperatures can significantly lower this barrier, resulting in an increased rate of diffusion [58]. This implies that as the temperature increases, gases and liquids diffuse more rapidly through polymers because more penetrant molecules have sufficient energy to overcome the activation energy barrier. Both diffusivity and solubility are described as temperature-dependent relationships using Arrhenius-type equations:

(7) $$D\left(T\right)=D_{0}\;\exp\;(-\frac{E_{D}}{RT})$$

(8) $$S\left(T\right)=S_{0}\exp\;(-\frac{∆H_{S}}{RT})$$

where *E_D_* is the activation energy for diffusion, ∆*H_S_* is the enthalpy change in solubility, *R* is the universal gas constant, and *T* is the absolute temperature [63].

The effects of temperature on the fraction-free volume void (FFV) of amorphous polymers have also been reported in the literature [58,66]. As the temperatures exceed *T_g_*, the amorphous regions reorganize. Consequently, the fractional volume of voids in the amorphous region increases linearly with temperature. This free volume is crucial for the diffusion of small molecules, such as gases, through a polymer. As a polymer ages, its FFV changes due to factors such as rearrangement of the polymer chains, environmental stress, or chemical reactions. The relation between the diffusion coefficient *D* of a molecule in a polymer and the FFV of the polymer can be expressed by Doolittle’s expression of the form [58]:

(9) $$D=A_{0}\exp(-\frac{B}{FFV})$$

where *A*_0_ and *B* are empirical constants.

The negative sign in Doolittle’s equation indicates an inverse relationship between the term $\frac{B}{FFV}$ and the diffusion coefficient *D*. As the FFV increases, the value of $\frac{B}{FFV}$ decreases, making the exponent less negative, which in turn causes the diffusion coefficient *D* to increase. Conversely, when FFV decreases, $\frac{B}{FFV}$ increases, resulting in a more negative exponent and a corresponding reduction in *D*.

The FFV is defined as the ratio of the free volume *V_f_* to the specific volume *V_sp_* of the polymer, which is expressed using [58]:

(10) $$\mathrm{FFV}=\frac{V_{f}}{V_{sp}}$$

where *V_f_* represents the free volume of the polymer, which is the space not occupied by polymer chains, while *V_sp_* is the specific volume, or the total volume, of the polymer per unit mass.

The free volume *V_f_* can be further defined using the expression [58]:

*V_f_* = *V_sp_* − 1.3*V_vw_* (11)

where *V_v_w* is the van der Waals volume, representing the volume occupied by the polymer chains. The *V_v_w* is typically constant for a given polymer.

In the specific case of CO_2_, which is a focus of gas diffusion in polymer, previous studies have shown its impact on thermoplastic materials [29,30,67]. The interaction of CO_2_ with polymer materials can significantly affect their properties due to the unique characteristics of CO_2_, especially when it is in a dense phase. CO_2_ can cause polymer matrices to swell as it behaves like a plasticizing agent, which reduces *T_g_* of the polymers, making them softer and more deformable at lower temperatures. Additionally, polymers may undergo chemical degradation when exposed to CO_2_, particularly if impurities such as oxygen, moisture, and other acidic gases are present. These impurities can lead to hydrolysis and oxidation reactions that deteriorate the polymer chains, diminishing their mechanical strength and altering their chemical resistance. Rapid gas decompression (RGD) is another concern, where absorbed CO_2_ expands rapidly during pressure drops, which causes a risk of structural integrity issues through cracks or ruptures. Moreover, at low temperatures, polymers and elastomers may lose flexibility through their weakened sealing capabilities, which are crucial for maintaining CCS integrity [29].

The absorption of CO_2_ is also influenced by the polarity and crystallinity of polymers. The mechanical properties of various polymer materials under different pressures and temperatures of CO_2_ exposure have been reported in the literature. Due to CO_2_ sorption, the stiffness of polymers reduces due to two underlying mechanisms. First, the absorbed CO_2_ can act as a plasticizer at the molecular level to increase the material’s flexibility and reduce its rigidity. Second, the accumulation of CO_2_ can expand the volume of a material due to swelling effects [30]. The interactions between polymers and CO_2_ can be elucidated by analyzing the Hansen solubility parameters. These parameters reflect the cohesive energy of molecules and indicate that similarities in the solubility characteristics of CO_2_-specific polymers can increase the absorption of CO_2_ and consequent swelling [30]. The effects of CO_2_ on PEEK in a supercritical phase (scCO_2_) result in modifications to its morphology and mechanical properties [67,68]. Further details on these effects can be found in PEEK aging in CO_2_.

### 4.2. Diffusion of Liquid Solvents in Polymers

The diffusion of solvents into polymers shares some similarities with gas diffusion but also has key differences. Both solvents and gases cause swelling in the polymer, but solvents have larger effects and interact more strongly with the polymer, leading to more swelling and plasticization [64]. The diffusion modeling of organic solvents and acids can be described based on Fick’s second law, and their solubility in polymers can be determined using Henry’s law, as explained in Section 4.1. In addition, liquid diffusion and solubility are both temperature-dependent and can be explained using Arrhenius relation. In general, the rate of diffusion of a liquid solvent is influenced by factors such as the available free space within the polymer, the temperature, and the properties of the solvent molecules.

The interaction of the solvent and polymer is largely influenced by their molecular structures and chemical compositions. The initial state of the polymer also plays a role, including its crystallinity (the orderly arrangement of molecules) and orientation (molecule alignment), and molecular weight distribution (the range of sizes of the polymer molecules). Additionally, external conditions such as temperature, pressure, and stress also impact how the solvent interacts with the polymer. These factors together determine how solvents affect polymers, which leads to changes like swelling, dissolution, or other alterations [64]. In addition to these physical and structural factors, chemical interactions and fluid absorption within polymers are also greatly affected by the polarity of the polymers and specific environmental conditions. Polymers undergo chemical interactions with various substances, such as acids and oils, based on their solubility parameters. These parameters are determined based on three key factors: dispersion (van der Waals forces), polarity (associated with dipole moment), and hydrogen bonding [65]. Furthermore, the polymer chains exhibit specific interactions with solvents that have similar chemical moieties (like dissolves like) [64].

The molecular size and polymer structure significantly influence substance permeability. A study conducted by Craster et al. [62] provided evidence that the polymer structure considerably impacts the rate of fluid permeability. Their study showed that the diffusion rates of sodium chloride (NaCl) and potassium chloride (KCl) in PEEK were higher than those in polyphenylene sulfide (PPS). At infinite dilution and 25 °C, the diffusion coefficients of NaCl and KCl were 1.6 × 10^−5^ and 1.9 × 10^−5^ cm^2^/s, respectively. KCl diffused more rapidly than NaCl in both PEEK and PPS, likely due to the smaller hydrated cation radius of potassium (0.66 nm) compared to sodium (0.72 nm). The study also reported that the diffusion coefficients of NaCl and KCl during permeation ranged from 0.9 × 10^−12^ cm^2^/s to 9.7 × 10^−12^ cm^2^/s during permeation, which highlights the influence of molecular size and polymer structure on the permeability [62].

**Figure 16:**
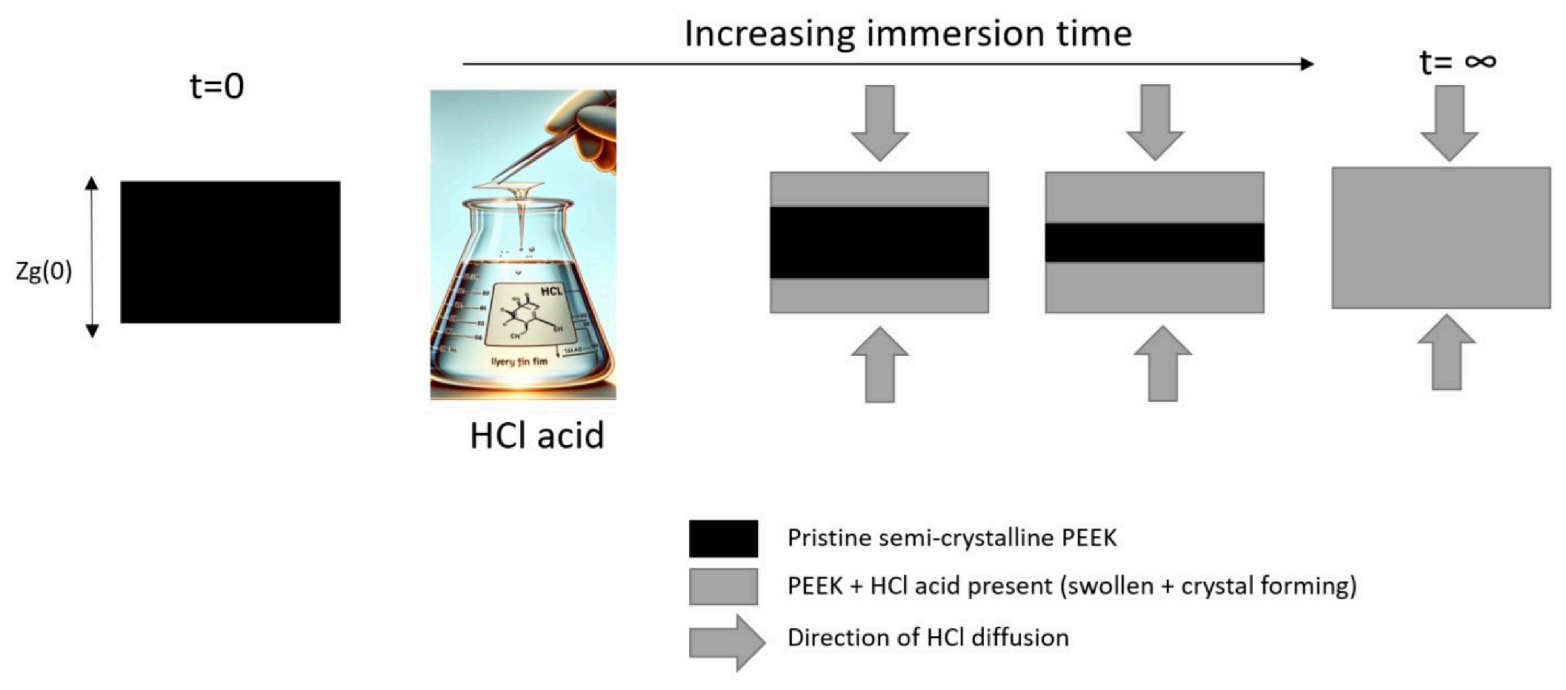
Cross-sections of films showing solvent diffusion kinetics with immersion time.

In solvent diffusion, some areas of the polymer may not become fully saturated. Wolf et al. studied the uneven swelling of PEEK film when exposed to a solvent, noting significant changes in film thickness perpendicular to the flat surface and a clear line in SEM images indicating where the solvent penetrated [69]. Figure 16 illustrates the transformation of the film as the solvent diffuses, with areas where the solvent has penetrated appearing thicker. The solvent plasticizes the polymer and moves faster through the flexible parts than through the non-flexible (unplasticized) regions, which creates a clear dividing line. Over time, the size of the rigid, glass-like areas of the film decreases as the solvent spreads, but the solvent never fully saturates the film [12]. This highlights that even when the sorption fronts meet in the middle of the film, the solvent absorption remains uneven. There is still a noticeable concentration difference between the solvent-saturated areas and the drier parts of the polymer, showing the complex nature of solvent diffusion in the material [12,64].

## 5. Aging of PEEK

The aging process can be divided into two categories: physical aging and chemical aging. Chemical aging involves changes in the molecular structure of PEEK, such as the breaking or formation of chemical bonds, leading to alterations in molecular weight [10]. This type of aging is often caused by environmental factors, such as chemical exposure, that degrade the polymer over time. Additionally, high temperatures in the presence of air can cause chemical reactions, like oxidation, which lead to crosslinking (forming new bonds between polymer chains), chain scission (breaking of polymer chains), and the formation of new functional groups of PEEK [70].

Physical aging, in contrast, involves changes in the structure and morphology of PEEK without altering its chemical composition. It occurs when polymers, especially in their glassy state, move towards a non-equilibrium state, which results in movements of their molecular chains. This physical aging of polymers may involve changes in properties such as free volume, *T_g_*, alterations in crystallinity, and melting temperature [10]. These changes help the polymer reach a more thermodynamically stable state. Odegard et al. [71] further classified aging into thermal aging, hygrothermal aging, and weathering, each with specific subcategories. They noted that physical aging in glassy polymers and epoxies typically leads to an increase in mass density and a decrease in molecular configurational energy when kept below their *T_g_*. This process, known as annealing, often results in reduced toughness, decreased viscoelastic response, and lower permeability, which impacts the durability of polymers. Physical aging can also occur alongside chemical and hydrothermal aging, which involves molecular degradation from environmental factors and moisture [71].

### 5.1. Thermal Aging

The mobility of polymer chains with temperature changes is crucial to understanding polymer behavior. As the temperature rises, thermal energy increases segmental mobility. In rigid, glassy materials below *T_g_*, molecular motion is limited to small rotations and noncooperative movements within the polymer backbone. Above *T_g_*, molecular motion becomes cooperative, making the material soft and rubbery. For crystallizable polymers like PEEK, heating above *T_g_* promotes crystallization [72].

Thermal history can be used to control the nanostructure of PEEK and its composites, particularly their crystallinity. The effects of thermal aging on PEEK depend significantly on its microstructure and degree of crystallinity, which influence its mechanical properties [73,74,75,76]. As crystallinity increases, PEEK’s shear and tensile strengths improve to enhance its resistance to harsh environments [77].

Expanding on this, some studies show that thermal treatment impacts PEEK’s morphology, especially in terms of crystallinity and spherulite size [78]. Isothermal crystallization produces more organized crystalline structures, which results in better fatigue and static performance than annealing crystallization [78]. These findings demonstrate that the degree of crystallinity has a greater influence on the mechanical properties of PEEK than the specific arrangement of its crystalline regions.

Sinmazçelik et al. [79] demonstrated the effects of thermal aging on the mechanical properties of unfilled PEEK and short fiber-reinforced PEEK composites. They found that thermal aging improves the flexural modulus and stiffness but reduces toughness, leading to increased brittleness and decreased impact resistance. The presence of reinforcing fibers, such as carbon or glass fibers, acts as nucleating agents, promoting the formation of transcrystalline layers that enhance the material’s stiffness by restricting molecular mobility. Thermal aging affects the crystallinity of PEEK differently depending on the conditions; shorter aging periods at lower temperatures promote crystal growth, while longer periods or higher temperatures lead to more perfect crystals but may reduce overall crystallinity due to matrix degradation. Overall, thermal aging has a more pronounced impact on unfilled PEEK, while fiber-reinforced PEEK shows less variability in crystallinity and more stable mechanical properties due to the influence of reinforcing fibers.

Zhu et al. [80] investigated the thermal aging behavior of PEEK by aging the specimens at temperatures between 170 and 310 °C for 14 days, followed by thermogravimetric analysis (TGA). Increasing the aging temperature progressively reduced both the maximum decomposition temperature and the temperature at 5% weight loss, indicating a gradual loss of thermal stability. The degradation process involved random scission of the ether and ketone bonds, followed by carbonyl bond decomposition, producing volatile species such as carbon monoxide (CO) and (CO_2_). Nevertheless, more than 45% of the original mass remained at 800 °C, confirming the excellent thermal stability of PEEK despite prolonged thermal aging [80].

Courvoisier et al. [70] evaluated the thermal degradation of PEEK in the rubbery state at temperatures between 180 and 320 °C and oxygen partial pressures from 0.21 to 50 bars. Using FTIR spectrophotometry, they identified degradation products such as benzoic anhydride, phenyl benzoate, fluorenone, phenols, and benzoic acid, which clarified the mechanisms of thermolysis and thermo-oxidation. At higher oxygen pressures, benzoic anhydride and phenyl benzoate were the predominant products, formed by the coupling of carboxyl and phenoxyl radicals. Additional analysis using DSC and micro-indentation showed that crosslinking was the dominant process over chain scission under all conditions, which increased crystallinity and Young’s modulus up to 290 °C. However, above this temperature, further crosslinking restricted molecular mobility, reducing both crystallinity and Young’s modulus. Similarly, Tsai et al. [81] found that at 450 °C, thermal decomposition of PEEK produces pyrolysates like 1,4diphenoxybenzene and 4-phenoxyphenol due to selective cleavage of chain ends and branches. Naffakh et al. [82] reported that degradation occurs at 400 °C in the presence of oxygen and is accelerated by branched-chain molecules. However, both studies found no evidence of dissociation at temperatures around 250 °C. Overall, these findings show that the thermal degradation of PEEK is influenced by temperature, oxygen pressure, and molecular structure. Crosslinking is the main process below 290 °C, while degradation becomes more rapid at higher temperatures.

Jin et al. [19] investigated the effect of isothermal crystallization on the lamellar microstructure of PEEK using small-angle X-ray scattering (SAXS). The results showed that the crystallization and melting temperatures strongly influenced the long period and lamellar morphology. The crystalline lamellae and amorphous layer thicknesses were approximately 3–5 nm and 10.5–12.5 nm, respectively. Higher melting and crystallization temperatures promoted a more ordered lamellar structure with a larger long period, whereas lower temperatures or quenching produced a less ordered, lower-crystallinity morphology [19].

**Figure 17:**
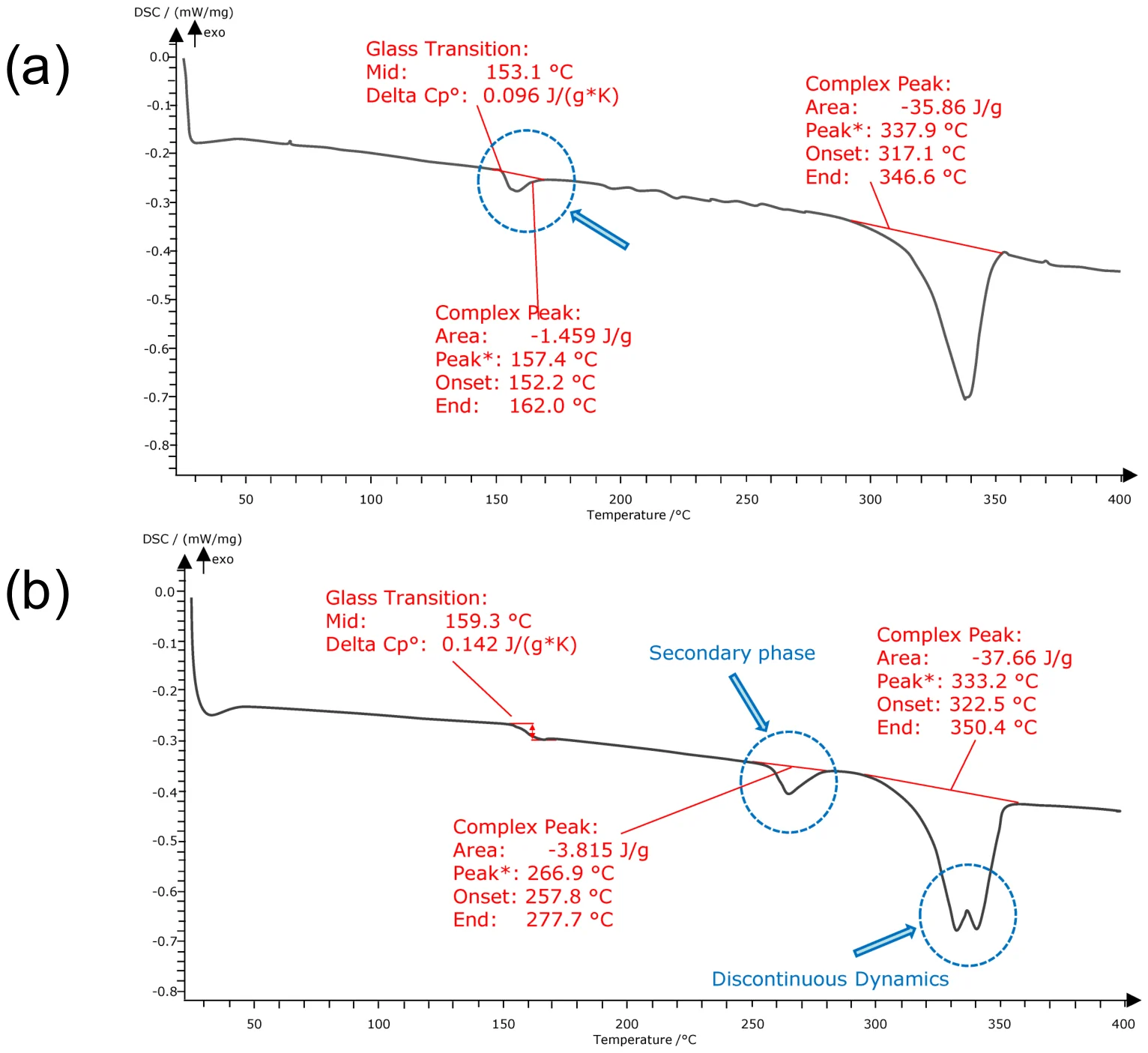
DSC results of (**a**) Pristine PEEK, and (**b**) PEEK subjected to thermal annealing for 2 h [83].

Compared with conventionally molded PEEK, the thermal aging behavior of fused filament fabrication (FFF)-printed PEEK has received increasing attention because the printing process generates a distinct microstructure, including interlayer interfaces, residual stresses, and variable crystallinity. In this study by Abbas et al. [83], PEEK specimens fabricated by FFF were subjected to thermal post-treatment by annealing at 250 °C for 2 h, followed by DSC analysis to investigate changes in crystallinity and thermal transitions. The first-heating DSC curve, as shown in Figure 17a of the as-printed PEEK, exhibits a *T_g_* of approximately 153.1 °C and a degree of crystallinity (DOC) of approximately 30%. A small relaxation endotherm is observed immediately after *T_g_*, indicating the presence of residual thermal stresses and a non-equilibrium amorphous phase generated during the FFF printing process [83].

After annealing, the DSC curve in Figure 17b for first heating shows that *T_g_* increases to approximately 159.3 °C, while the DOC increases slightly to about 31–32%.

The secondary melting peak appears at approximately 267 °C, indicating the formation of secondary crystals during thermal treatment. These results demonstrate that annealing promotes secondary crystallization and relieves residual stresses, leading to improved molecular ordering and reduced chain mobility [83]. The small changes in the overall degree of crystallinity, together with the secondary endothermic peak, suggest that annealing mainly reorganizes and stabilizes the existing crystalline regions, rather that significant increasing the total amount of crystals in PEEK [83].

### 5.2. Water Aging

PEEK has a relatively low level of sorbed water content. Density measurements were performed both prior to and subsequent to the samples achieving equilibrium. Based on the findings, it is noteworthy that the densities of the saturated samples align with those of the initial dry samples, although the samples absorbed approximately 0.5% of water. The solubility of water increased from 0.44 weight percent at 35 °C to 0.55 wt% at 95 °C [84]. Grayson and Wolf also noted no alteration in matrix morphology during water sorption and desorption [84].

The sorption of liquid water and water vapor in amorphous and semicrystalline PEEK was examined by Mensitieri et al. [85]. The observed behavior of amorphous PEEK films can be attributed to their low water vapor activity, which is consistent with Fickian diffusion. At elevated levels of water vapor activity, the amorphous samples exhibited unconventional diffusion mechanisms by combining Fickian diffusion with a first-order relaxation term [85]. This indicates greater interaction with high water vapor activity, leading to higher diffusion rates and possibly more complex behavior. Their investigation revealed that the process of water sorption in semicrystalline PEEK adheres to the classical Fickian mechanism across all temperature ranges. Mensitieri et al. revealed that the water intake at equilibrium is constant across different temperatures, maintaining an average value of 0.48%. The water sorption does not affect the microstructure of PEEK [85].

In another investigation conducted by Porto et al. [86], the aging behavior of PEEK was assessed under various environmental conditions such as air, water with nitrogen, water with air, and acidic water (pH 4) for 90 days at elevated temperatures of 120 °C, 140 °C, and 160 °C. They revealed that PEEK maintained its physical integrity, as evidenced by negligible water absorption and mass loss. Thermal stability as assessed through thermogravimetric analysis remained largely consistent across all aging media, with minimal deviations in thermal degradation onset. DSC indicated no significant alterations in thermal transition temperatures, suggesting the absence of internal degradation within the polymer. An increase in crystallinity, as determined by XRD and DSC, was observed. This increase can be attributed to thermal annealing effects at temperatures above *T_g_* of pure PEEK. The evaluation of mechanical properties revealed marginal changes in Young’s modulus, signifying consistent stiffness. However, variations in tensile strength and elongation at break (EAB) were more pronounced, particularly in aqueous environments enriched with air and acidic solutions. These findings underscore the resilience of PEEK in maintaining its stiffness and thermal properties, while also highlighting the sensitivity of its tensile characteristics to specific aging conditions [86].

**Figure 18:**
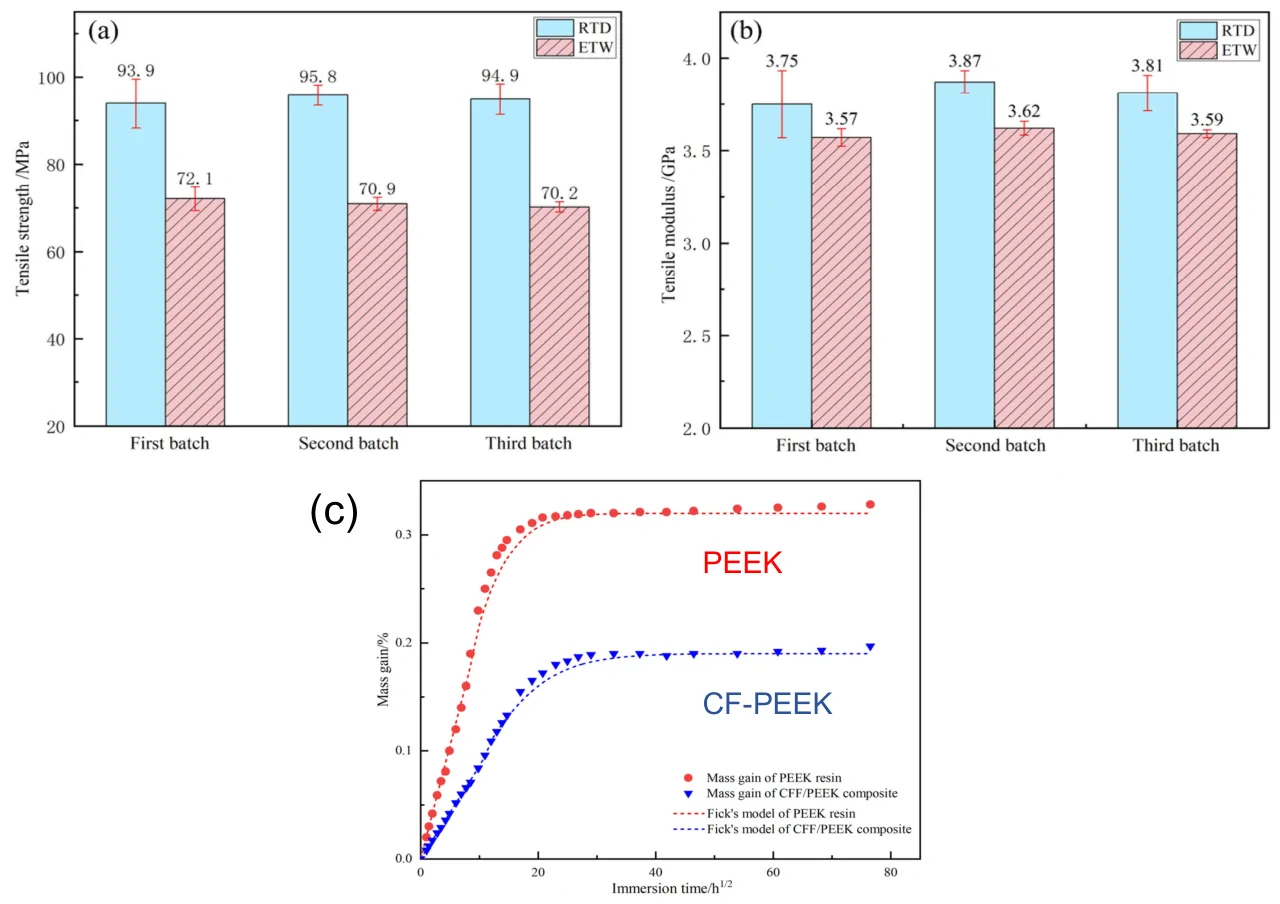
Effect of hygrothermal aging on the tensile properties of PEEK resin under dry room temperature (RTD) and equilibrium temperature-wet (ETW) conditions: (**a**) Tensile strength, (**b**) Tensile modulus, and (**c**) Water absorption behavior and Fickian diffusion model fitting of PEEK resin and CFF/PEEK composite [87].

Xu et al. [87] evaluated the effect of hygrothermal aging on PEEK and CF/PEEK composites. The specimens were first dried and then exposed to 70 °C and 85% relative humidity until equilibrium moisture absorption was reached, following ASTM D5229 [88]. The mechanical properties were subsequently evaluated under dry room temperature (RTD) and equilibrium temperature-wet (ETW) conditions. Hygrothermal aging significantly reduced the tensile strength of neat PEEK, whereas the tensile modulus showed only a slight decrease, as demonstrated in Figure 18a,b [87]. This behavior was attributed to moisture-induced plasticization of the amorphous regions, which increased molecular chain mobility and reduced the load-bearing capacity while having only a limited effect on the stiffness of the semi-crystalline structure [87].

The moisture absorption behavior of PEEK resin and CFF/PEEK composites was well described by the Fickian diffusion model during the initial stage, where moisture uptake increased linearly with the square root of time as illustrated in Figure 18c [87]. As moisture absorption progressed, the experimental data deviated from the Fickian model due to the combined effects of temperature and humidity, which accelerated water diffusion through the resin and internal defects within the composite. The moisture absorption rate gradually decreased until equilibrium was reached, with equilibrium moisture contents of approximately 0.32% for PEEK resin and 0.19% for the CFF/PEEK composite, indicating the improved moisture resistance of the composite [87].

### 5.3. Chemical Aging

This section examines the aging of PEEK in organic solvents and acids, which can affect its crystallinity, trigger chemical reactions with functional groups, and alter intermolecular forces. These effects are based on various studies conducted on PEEK, which demonstrate that exposure to different chemical environments can lead to significant changes in its structural and chemical properties.

The exposure of PEEK to solvents can affect its properties in several aspects, including dimensional changes, mechanical property alterations, surface degradation, and chemical changes [12]. These changes may occur due to a physical phenomenon, such as the penetration of solvent molecules into the PEEK matrix, leading to swelling and plasticization, or a chemical phenomenon, where reactions occur within the PEEK structure [10,11]. The chemical structure of PEEK contributes to its inherent resistance to many solvents. It comprises repeating aromatic ketone units linked via ether linkages, which offers excellent thermal stability and resistance to oxidative and hydrolytic degradation [16,89]. However, the presence of the aromatic backbone makes PEEK susceptible to attack from certain classes of solvents, especially those with high polarity or specific functional groups [12,16]. Several accelerated aging studies have been performed to evaluate the thermal and chemical resistance properties of PEEK, mostly on exposure to water rather than organic solvents and hydrocarbons [84]. For example, Mensitieri et al. [85] demonstrated that water sorption in semicrystalline PEEK follows a classical Fickian description over the entire range of temperatures. Arzak et al. [90] demonstrated that amorphous PEEK absorbs methylene chloride faster than highly crystalline PEEK (30% crystallinity), owing to the impermeability of its crystalline phase.

Particularly, for aging of PEEK in acids, most of the available studies have focused on the effect of different acids on the surface characteristics and microstructure response [91]. For instance, Makanantachote et al. [92] investigated the effects of sulfuric acid aging on PEEK to introduce sulfonate functional groups, which enhances the surface roughness and adhesion with dental adhesive for medical use. They concluded that the change in surface roughness due to aging was insufficient to achieve good interlocking between the PEEK and adhesive, and thus insignificant enhancement in the strength was observed. Similarly, Zhou et al. [93] evaluated the impact of sulfuric and hydrofluoric acid treatments on PEEK’s surface and bonding properties. Sulfuric acid created a porous surface, enhancing adhesion, while hydrofluoric acid had minimal effect on surface texture. SEM analysis confirmed sulfuric acid’s efficacy in modifying PEEK’s topography, indicating its potential to improve adhesive bonding capabilities and surface roughness.

Molecular mobility can also be influenced by chemical and thermal environments. In addition, interactions between chemical species promote the structural rearrangement of PEEK chains. Stuart and Williams [65,94] compared solvent and thermal effects on amorphous PEEK using Raman spectroscopy and found that the carbonyl stretching mode of PEEK when exposed to tetrachloroethane (TCE) matched with those of the polymer unexposed to the solvent but heated to approximately 250 °C. PEEK molecular relaxation processes induced by solvents were comparable to those induced thermally at temperatures well above its *T_g_*. In other words, we can observe both thermally activated and chemically activated chain mobility, and in some cases, developed an equivalence principle between these two stimuli.

Ahmad et al. [89] investigated the chemical and thermal behavior of amorphous PEEK in various organic solvents. After two months of immersion, amorphous PEEK sheets were analyzed using FTIR, TGA, and DSC. The study found that toluene, benzene, and tetrahydrofuran (THF) altered the chemical structure of the polymer. FTIR results indicated no significant surface chemical changes in most solvents except for THF, which introduced a nonbonded O-H group due to absorbed water. TGA revealed that chloroform and acetone reduced the polymer’s maximum service temperature, and various solvents also induced polymer crystallization [89]. In addition, solvents with saturated ring structures similar to PEEK, such as benzene and toluene, were also found to promote polymer enlargement and crystallization [12].

In another study of aging in solvents, Mensitieri et al. [95] observed that PEEK exhibited significant interactions with some organic solvents. The thermoplastic matrix exhibited a robust interaction with methylene chloride (CH_2_Cl_2_), mostly attributed to the hydrogen bonding and solubility features [95]. The aforementioned interactions led to typical sorption characteristics of CH_2_Cl_2_ in PEEK samples with varying degrees of crystallinity. Moreover, the solvent permeating the material plasticized the noncrystalline structure, thereby reducing the free energy of the crystalline arrangement. The occurrence of this event led to the observation of solvent-induced crystallization during the sorption of CH_2_Cl_2_ in PEEK, even in samples with high initial crystallinity [95].

**Figure 19:**
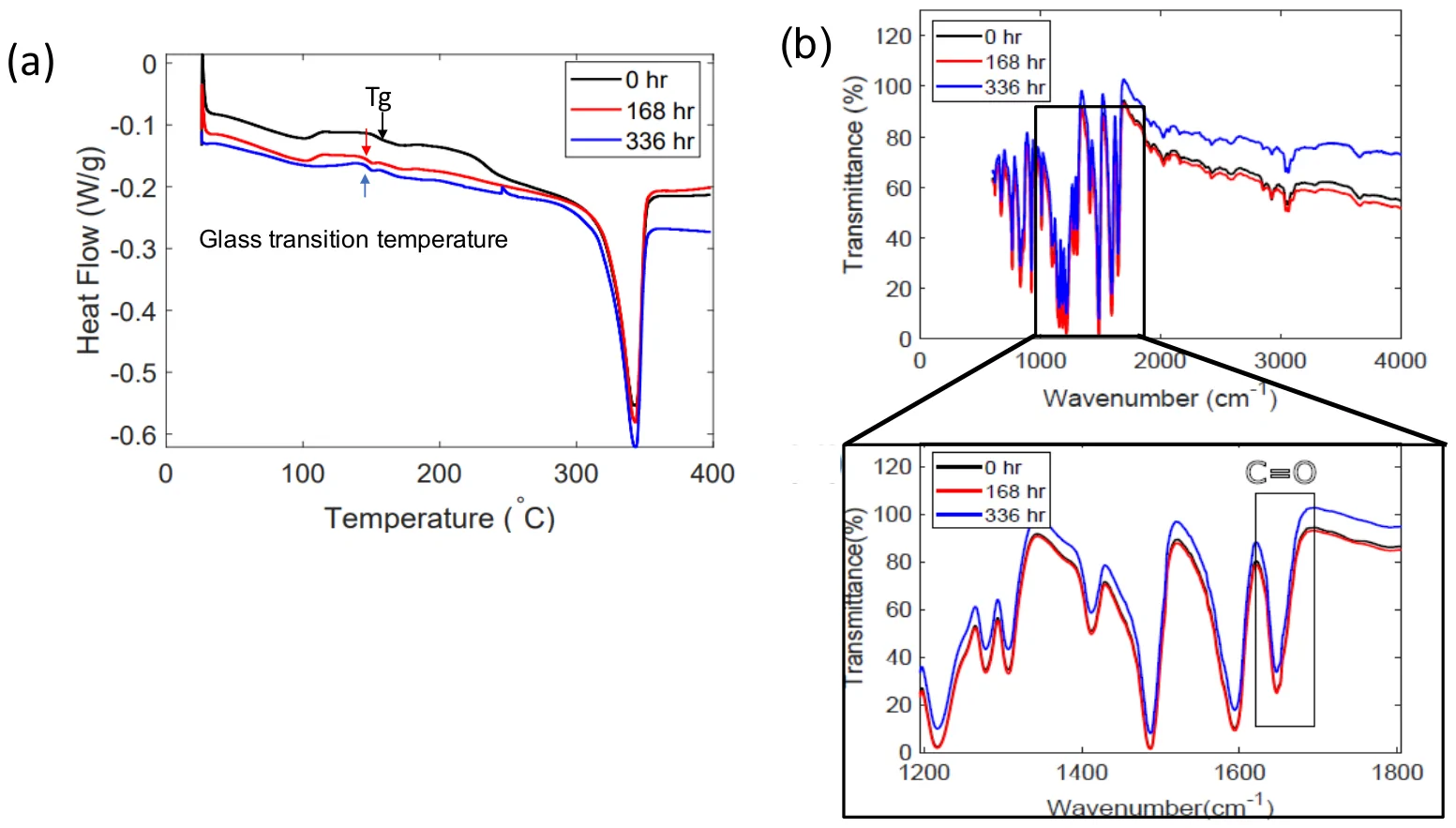
PEEK’s microstructure changes after aging: (**a**) DSC analysis of PEEK aged in HCl at 100 °C for 336 h, and (**b**) FTIR analysis of PEEK samples aged in HCl at 100,100 °C for 336 h [96].

In another study, Badeghaish et al. [96] investigated the effect of aging PEEK in 37% HCl at 100 °C for 336 h and evaluated its thermal and chemical response before and after exposure. Figure 19a shows the DSC curve, where a slight reduction in the glass transition temperature (*T_g_*) was observed, indicating mild plasticization of the amorphous phase due to HCl diffusion. In addition, a small secondary crystallization peak appeared, accompanied by a modest increase in the degree of crystallinity (approximately 2–4%, depending on the film thickness). These findings suggest that HCl exposure promotes physical chain rearrangement and secondary crystallization without causing significant chemical degradation of the PEEK matrix [96].

Figure 19b presents the FTIR spectra of PEEK before and after exposure to HCl. No significant changes were observed in the spectra, with all characteristic absorption peaks remaining unchanged. Only a slight variation in the intensity of the carbonyl peak was detected, indicating a weak physical interaction between HCl and PEEK. These results confirm that HCl exposure does not alter the chemical structure of PEEK or cause significant chemical degradation [96].

### 5.4. CO_2_ Aging

Steel is the primary material for transporting pressurized CO_2_, but its susceptibility to corrosion poses challenges [29]. In carbon capture and sequestration (CCS), polymers are crucial throughout the CO_2_ value chain, especially in transport [97]. However, research on polymers for components like gaskets and pipes is limited, and their use raises concerns about chemical compatibility, permeability, thermal performance, and resistance to RGD [29]. Ansaloni and Khalid have reported on the effects of CO_2_ on thermoplastic materials such as (HDPE, PP, PA12, PTFE, PVDF, and fluoro-elastomer) [3,30]. They demonstrated that CO_2_ expansion can irreversibly alter the properties of polymers. The polarity and crystallinity of polymers also affect CO_2_ absorption. HDPE, PTFE, and PA absorbed smaller amounts of CO_2_ (1%) at a pressure of 6.5 MPa [30].

In engineering thermoplastics used in vessels and pipes, the presence of crystallinity can restrict CO_2_ sorption and volumetric swelling within the polymer matrix, compared to what is observed in amorphous thermoplastics or elastomers [30].

Gases like CO_2_ pose challenges in the pipeline and can significantly alter the physical and chemical properties of polymers by incorporating into the matrix and potentially inducing physical and chemical changes, such as chain scission, which might degrade mechanical properties [3,30,97]. The absorption effects of CO_2_ on polymers were thoroughly discussed in (Section 4.1). The plasticization and crystallization of amorphous PEEK in CO_2_, which is a supercritical fluid [67,68], modify the polymer’s morphology and mechanical properties.

Mohammed et al. [55] analyzed the physical properties of PEEK exposed to severe oilfield conditions. PEEK was subjected to aging processes at high temperatures using various gases, including a combination of methane and CO_2_, and CO_2_ alone. They revealed that exposure to gases causes irreversible changes in the extent and shape of the crystalline structure. Depending on the nature of the gas, this growth in crystallinity can be overturned if widespread plasticization of the amorphous phase is permitted. A comparison of DSC, DMTA, and PALS data shows a coherent view of these changes that are not immediately apparent in terms of density and volume changes. Moreover, the crystallinity changes induced by the initial thermal annealing persist unless the disordered regions are highly plasticized [55]. DMTA revealed that high-temperature aging did not considerably alter the *T_g_* of PEEK. This indicates that while the crystalline portion of PEEK increased, signifying a more orderly structure, the amorphous part remained largely unaffected. A notable effect was observed when PEEK was exposed solely to CO_2_, which caused significant plasticization of the material and improved its flexibility. This was evident from the emergence of two distinct peaks in the tan delta measurements and a reduction in the bending modulus at lower temperatures. However, the plasticization effect was reversible. Upon heating the samples, the *T_g_* and bending modulus of PEEK increased beyond the original values of the unaged material. Interestingly, despite the reversibility of the plasticization, the changes in the crystalline structure of PEEK induced by the aging process were permanent [55].

**Figure 20:**
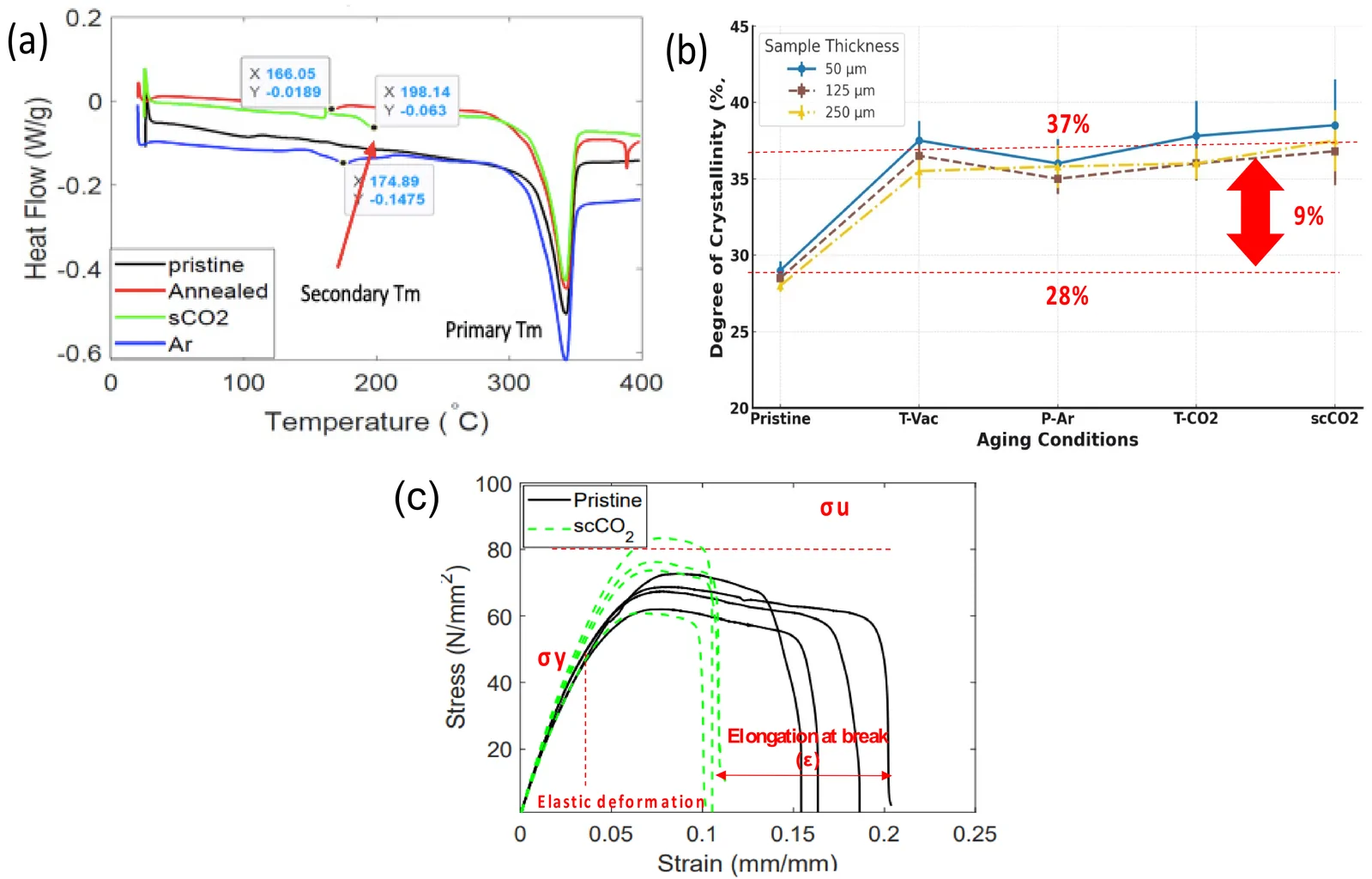
PEEK’s microstructure changes after scCO_2_ aging: (**a**) DSC analysis of PEEK samples in various conditions, (**b**) degree of crystallinity of PEEK with different thicknesses under scCO_2_, and (**c**) stress–strain curve of PEEK samples under scCO_2_ aging conditions [96].

In a recent study on the effect of supercritical CO_2_ (scCO_2_) on PEEK morphology, Badeghaish et al. [96] exposed PEEK thin-film specimens to scCO_2_ and other gases at 150 °C and 100 bars for 168 h using a 500 mL high-pressure stainless-steel vessel. The study compared the effects of different aging environments on the thermal and mechanical properties of PEEK, including supercritical CO_2_ (150 °C, 100 bar), argon (150 °C, 100 bar), vacuum (150 °C), and CO_2_ in the gas phase (150 °C, 30 bar). This comparison helped distinguish the individual effects of temperature, pressure, and the CO_2_ environment on the aging behavior of PEEK. As shown in Figure 20a, all aged samples exhibited a secondary melting peak, indicating microstructural rearrangement during aging. Among the aging conditions, scCO_2_ produced the highest secondary melting temperature and the largest increase in crystallinity, reaching approximately 37% with different thicknesses and gas conditions compared with about 28% for pristine PEEK, as shown in Figure 20b. These results suggest that scCO_2_ promotes secondary crystallization and the formation of more stable crystalline structures through physical interactions without causing chemical degradation. Moreover, the results showed that temperature is the main factor driving crystallization during aging, while the effect of the different gases is smaller and dissolved CO_2_ increase chain mobility, which can further promote chain rearrangement and crystallization. Although the plasticizing effect of CO_2_ is mostly reversible after the gas escape, the resulting changes in the crystalline structure can remain. Hence, the change in degree of crystallinity almost similar with different gas conditions [96].

Figure 20c compares the tensile properties of pristine and aged PEEK. Both the yield strength and ultimate tensile strength increased after aging, with the greatest improvement observed for the scCO_2_-aged samples (approximately 25% and 8%, respectively). In contrast, the elongation at break decreased, indicating reduced ductility due to the higher crystallinity and more ordered polymer structure [96].

In addition, Mohammed et al. [55] demonstrated that PEEK aged in 100% CO_2_ at 200 °C exhibited the lowest increase in melting temperature (*T_m_*) with a small increase in the crystallinity compared to other tests in (N_2_ and CO_2_–CH_4_). It is anticipated that the CO_2_ acts as a plasticizer within the matrix, potentially inhibiting or reversing the enhancements in crystalline structure typically achieved through high-temperature annealing. Mixtures of CO_2_ and methane (CH_4_) appear to facilitate matrix reorganization, enhancing crystallinity and *T_m_*, with the most significant restructuring observed at 200 °C. The effectiveness of CO_2_ in this role is likely due to its larger molecular size (2.34 °A) compared to nitrogen (0.75 °A) and methane (2.17 °A), which allows it to more effectively control and modify the crystallinity level [55].

In a separate study, Wang et al. [98] investigated the CO_2_ sorption and diffusion behavior of three PEEK films with different chemical structures, namely 3H-PEEK, 3F-PEEK, and 6F-PEEK. The films were exposed to CO_2_ at different temperatures and pressures, and the sorption/desorption kinetics were analyzed using a Fickian diffusion model. The diffusion behavior agreed well with the Fickian model at low pressures but gradually deviated at higher pressures. Replacing hydrogen with CF_3_ groups increased the fractional free volume of the polymer, leading to higher CO_2_ solubility and diffusivity. As a result, the fluorinated PEEK grades showed higher CO_2_ permeability than 3H-PEEK, demonstrating the strong influence of polymer structure on gas transport [98].

In the study on PEEK’s crystallization with supercritical scCO_2_, Bologna et al. [99] conducted experiments under varying temperatures and pressures and reported relevant findings. At 80 °C and a pressure of 150 bar, they observed no change in PEEK’s crystallinity, attributed to the low solubility of CO_2_ at this temperature, which is insufficient for inducing crystallization. However, a notable increase in crystallinity was observed at 140 °C, surpassing the changes attributed solely to temperature. The study also highlighted the crucial role of pressure: at 150 bar, the increase in crystallinity was modest, but at 300 bar, there was a significant jump in crystallinity to 14.6% and 21.1%, respectively. This underlines that while the temperature has a notable impact, the increased pressure substantially influences PEEK’s crystallization when exposed to scCO_2_. They also highlighted the intricate interplay of temperature and pressure in inducing the structural changes in PEEK [99]. For semi-crystalline PEEK, no significant effect of CO_2_ on the degree of crystallinity and temperature was observed at 150 bar. However, at 300 bar, the CO_2_ effect became apparent, and the crystallinity increased to 30.7%.

Further research work by Wang et al. [100] investigated the effect of CO_2_ on the crystallization and thermal transition behavior of PEEK using DSC and DMA. The results showed that CO_2_ reduced both the cold crystallization temperature and *T_g_*, indicating increased molecular chain mobility. The changes became more pronounced with increasing CO_2_ uptake and exposure time. At low annealing temperatures (60–90 °C), two cold crystallization peaks were observed, whereas these peaks gradually disappeared after annealing at 100–160 °C, indicating that crystallization was nearly complete. These results demonstrate that both CO_2_ exposure and annealing temperature strongly influence the crystallization behavior and thermal properties of PEEK [100].

**Figure 21:**
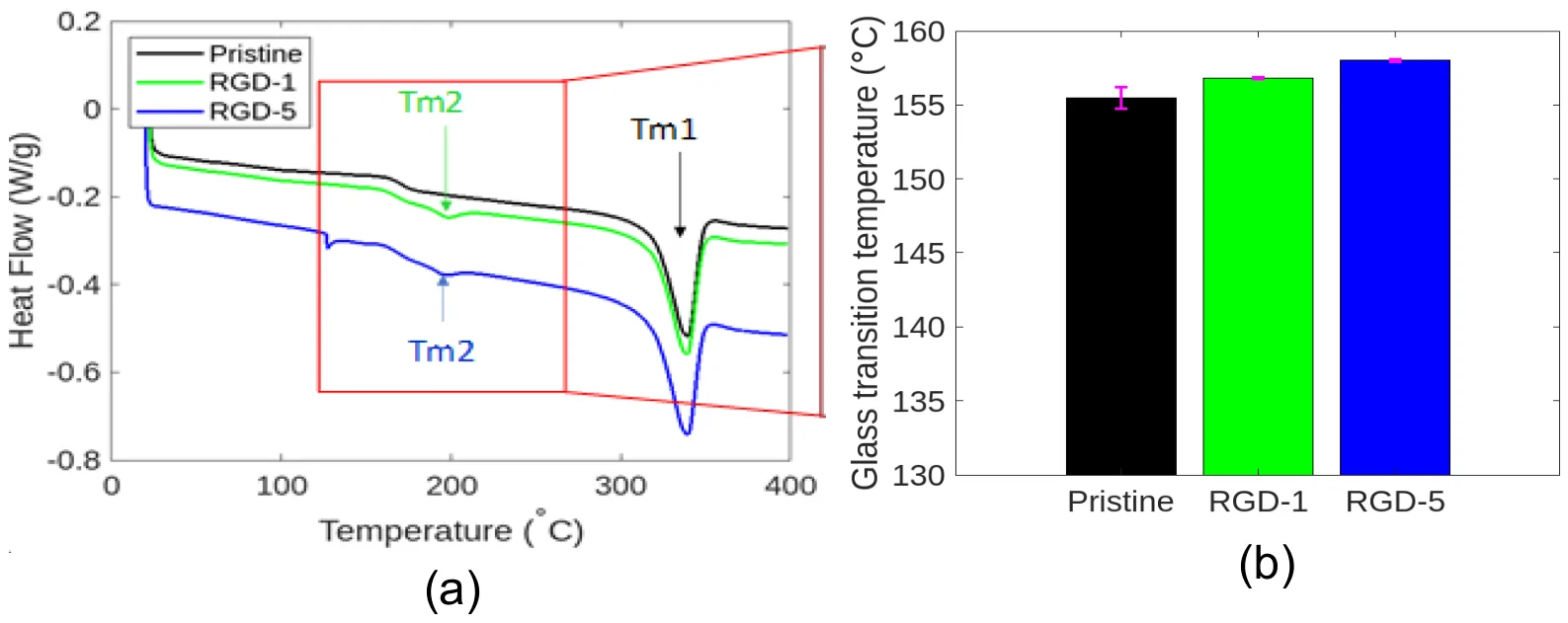
Characterization of 3D-printed PEEK before and after aging in supercritical CO_2_ (150 °C, 100 bar): (**a**) DSC thermograms, (**b**) bar-chart summary for Tg analysis for different conditions [101].

Regarding the transition from molded PEEK aging to 3D-printed PEEK, the study by Badeghaish et al. [101] evaluated the impact of scCO_2_ aging and rapid gas decompression (RGD) on the morphology of 3D-printed PEEK. 3D-printed PEEK specimens were aged in scCO_2_ at 150 °C and 100 bars. Two aging conditions were investigated: a single exposure for 120 h (RGD-1) and five exposure cycles of 24 h each, giving a total aging time of 240 h (RGD-5). After each aging cycle, the pressure was rapidly released to simulate RGD. The DSC results in Figure 21a showed a secondary crystallization peak at about 195 °C after both RGD treatments. The degree of crystallinity increased following scCO_2_ exposure, from 22.71% for pristine PEEK to 24.92% after one RGD cycle and 25.60% after five RGD cycles. These results indicate that repeated exposure to scCO_2_ promoted additional crystallization in the 3D-printed PEEK specimens [101]. Figure 21b showed that the Tg of pristine 3D-printed PEEK was 155.48 ± 0.71 °C. After the first rapid gas decompression cycle (RGD-1), Tg slightly increased to 156.84 ± 0.05 °C, indicating no significant change. After five cycles (RGD-5), Tg further increased to 157.84 ± 0.09 °C. This indicates that the aging process enhances molecular mobility and increases crystallinity, leading to an increase in Tg, without any plasticization effects [101].

**Figure 22:**
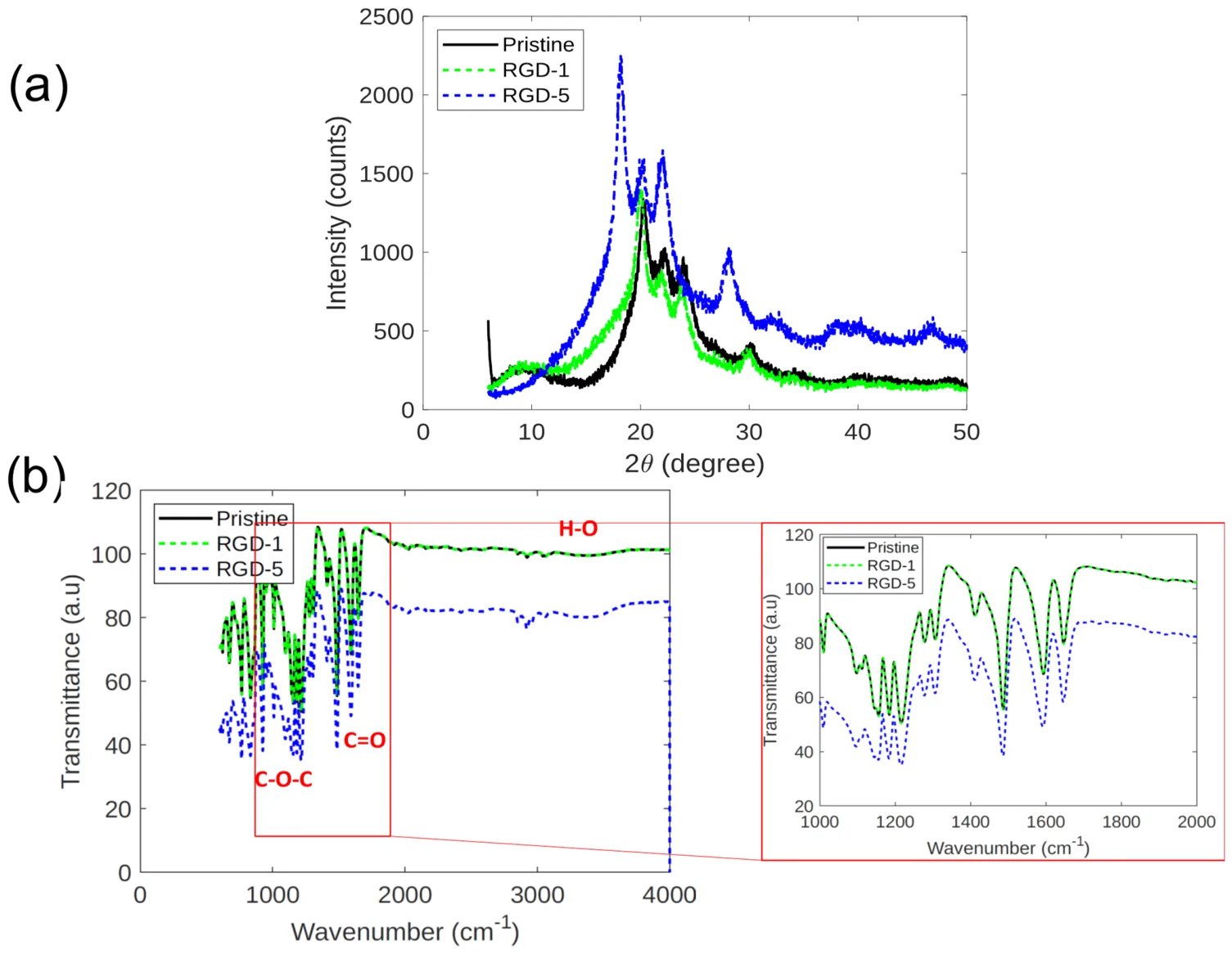
Characterization of 3D-printed PEEK before and after aging in supercritical CO_2_ (150 °C, 100 bar): (**a**) XRD patterns showing the degree of crystallinity, and (**b**) FTIR spectra showing the chemical structure before and after RGD aging conditions [101].

XRD analysis confirmed the DSC results, with slightly lower crystallinity values. The crystallinity increased from 17.4% for pristine PEEK to 20.4% for RGD-1 and 21.6% for RGD-5. The difference between the pristine and aged samples was small as shown in Figure 22a. However, FTIR analysis was performed to evaluate the effect of scCO_2_ aging on the chemical structure of 3D-printed PEEK after exposure to 150 °C and 100 bar under conditions RGD-1 and RGD-5. The FTIR spectra in Figure 22b shows no significant changes after aging, with no new absorption peaks or disappearance of the characteristic PEEK bands. Only small changes in peak intensity were observed, indicating minor physical interactions between scCO_2_ and PEEK. These results confirm that the aging of scCO_2_ did not alter the chemical structure of the 3D-printed PEEK, and all changes are physical [101].

While the aging behavior of conventionally processed PEEK is primarily governed by CO_2_ diffusion through the amorphous phase and the resulting changes in free volume and crystallinity [95,98,100], the response of AM PEEK is further influenced by its unique printed microstructure. AM PEEK contains interlayer interfaces, residual voids, and anisotropic raster architectures that can serve as preferential pathways for CO_2_ transport and modify local stress distributions during gas sorption and desorption. Consequently, although scCO_2_ aging induces only minor changes in the bulk thermal and mechanical properties of AM PEEK, localized microstructural evolution at interlayer regions and voids may influence fracture resistance by facilitating crack initiation and propagation [39,49]. However, the bulk thermal properties and mechanical strength remain largely unchanged, indicating limited degradation of the polymer matrix [39]. In contrast, fracture resistance may decrease because localized microstructural changes at interlayer regions and voids promote crack initiation and propagation [39]. These observations indicate that the long-term durability of AM PEEK in CO_2_-rich environments is governed not only by polymer morphology but also by defects introduced during the printing process. Further studies are therefore needed to establish the relationship between printing parameters, microstructural heterogeneity, CO_2_ transport, and long-term performance under representative service conditions. Table 2 summarizes the reported aging conditions and their impact on morphology and mechanical response.

Compared with other engineering thermoplastics commonly used in the oil and gas industry, such as PA6 and HDEP [103,104,105], PEEK exhibits superior resistance to CO_2_-induced plasticization, swelling, and mechanical degradation. This behavior is attributed to its rigid aromatic backbone, high crystallinity, and high glass transition temperature (143 °C), which limit CO_2_ diffusion and sorption. Under ambient conditions, PEEK typically absorbs only 0.1–0.3 wt% CO, increasing to approximately 0.5–2 wt% under supercritical CO_2_ (40–80 °C, 80–150 bar), resulting in less than 1–2% volumetric swelling and generally less than 10% reduction in tensile strength and elastic modulus, with most properties recovering after depressurization. In contrast, PA6 exhibits substantially higher CO_2_ affinity due to its polar amide groups, with CO_2_ uptake ranging from 1 to 3 wt% at low pressure and 6–15 wt% under supercritical conditions [106]. Consequently, PA6 can experience 5–15% volumetric swelling, 15–40% reductions in tensile strength, and 20–60% decreases in elastic modulus, with repeated pressure cycles potentially causing permanent dimensional changes [107]. HDPE demonstrates intermediate behavior. Although its nonpolar molecular structure results in lower CO_2_ solubility than PA6, it is more susceptible to gas-induced swelling than PEEK because of its lower stiffness and melting temperature. Typical CO_2_ uptake in HDPE ranges from 0.5 to 1.5 wt% under supercritical conditions, accompanied by approximately 2–6% volumetric swelling and 10–30% reductions in mechanical properties, depending on crystallinity, pressure, temperature, and exposure duration [58,108]. These differences explain why PEEK is preferred for high-value components operating in high-pressure CO_2_ environments, such as seals, valve seats, electrical feedthroughs, and downhole tools, where dimensional stability and long-term mechanical integrity are critical, whereas PA6 and HDPE are generally limited to less demanding applications where cost considerations outweigh extreme environmental performance.

Although numerous studies have investigated the aging behavior of PEEK under thermal, hydrothermal, chemical, radiation, and high-pressure gas environments, comparatively few have focused on predicting its long-term service life. In engineering practice, lifetime assessment relies on accelerated aging methodologies, with the Arrhenius model being the most widely adopted approach to correlate degradation rates measured at elevated temperatures with those expected under service conditions. Other predictive methods, including time–temperature superposition, diffusion-limited oxidation models, and creep and stress-relaxation analyses, are also employed depending on the dominant degradation mechanism. The remaining service life of PEEK components is typically evaluated by monitoring the evolution of key material characteristics, including tensile strength, elastic modulus, elongation at break, creep resistance, fatigue life, fracture toughness, hardness, and wear rate, together with changes in mass, dimensional stability, crystallinity, molecular weight, glass transition temperature (Tg), melting temperature (Tm), oxidation index (measured by FTIR), thermal stability (TGA), and chemical structure. By establishing degradation kinetics for these characteristics, accelerated aging data can be extrapolated to predict the long-term durability of PEEK under representative oil and gas service conditions, where design lifetimes commonly exceed 20–30 years, which is scarce in the literature.

## 6. Conclusions and Research Gap

PEEK is a semi-crystalline thermoplastic known for its strength, rigidity, chemical resistance, and biocompatibility, making it promising for critical oil and gas (O&G) applications. While PEEK is well-suited to harsh chemical environments, the full impact of aggressive conditions on its morphology and mechanical properties remains unclear. This review highlights the need to better understand PEEK’s aging processes, especially under the demanding conditions found in O&G operations. Most existing studies focus on short-term effects and standardized tests (e.g., ISO 23936) [109] that primarily assess macro-structural properties. To accurately predict PEEK’s long-term durability, future research should integrate these tests with microstructural characterization (such as changes in crystallinity, free volume, and diffusion behavior). Based on the current literature, the following research gaps have been identified:

- Uncertainty about PEEK’s durability in extreme downhole environments (e.g., high temperatures, high pressures, acidic conditions like HCl).
- Lack of experimental studies simulating realistic O&G operational conditions to validate PEEK’s performance in high-pressure and high-temperature supercritical CO_2_.
- Need for long-term aging studies to simulate prolonged exposure to aggressive environments and better assess PEEK’s durability.
- Limited evaluation of the erosion and wear resistance of both extruded and 3D-printed PEEK under conditions such as sand production.
- Integration of experimental work with numerical simulations (e.g., COMSOL, molecular dynamics) is needed to understand PEEK’s behavior in scCO_2_ and acidic media, and to assess the effects of voids and diffusion in 3D-printed PEEK.

## Figures and Tables

**Table 1:** Comparison of engineering thermoplastics commonly used in oil and gas applications [30].

Property	HDPE	PP	PA11/12	PVDF	PTFE	PEEK
Glass transition, *T_g_* (°C)	−125	−10	45	−35	121	143
Max. service temp. (°C)	100	110	120	150	260	260
Mechanical strength	M	M	G	G	M	E
Acid resistance	G	G	M	E	E	E
Hydrocarbon resistance	G	G	G	E	E	E
CO_2_ compatibility	G	G	M	G	E	E
CO_2_ uptake	L	L	M	LM	VL	VL
HPHT suitability	L	L	M	G	G	E
Relative cost	L	L	M	H	H	VH

Abbreviations: E = Excellent; G = Good; M = Moderate; L = Low; LM = Low–Moderate; VL = Very Low; H = High; VH = Very High.

**Table 2:** Summary of PEEK aging behavior under different environmental conditions.

Condition	Main Mechanism	Morphology	Thermal		Mechanical	Ref.
Thermal	Physical aging; secondary crystallization	*X_c_* ↑; Lam. ↑	*Tg*	↑; *T_m_* ≈	*E,H* ↑; *ε_b_* ↓	[19,79,81,82]
Water/humidity	Moisture diffusion	Swelling ≈; *X_c_* ≈		*T_g_* ≈	Properties ≈	[84,85]
Acids/solvents	Diffusion; plasticization	*X_c_* ↑; swelling ↑	*Tg*	↓; *T_m_* ≈	*σ* ≈; *ε_b_* ↓	[86,89,90,102]
CO_2_/scCO_2_	Gas sorption; secondary crystallization	*X_c_* ↑; FV ↓	*Tg*	↑; *T_m_* ≈	*E,H* ↑; *σ* ≈	[30,39,97,98,99,100]

Note: *X_c_* = crystallinity; Lam. = lamellae; FV = free volume; *T_g_* = glass transition temperature; *T_m_* = melting temperature; *E* = modulus; *H* = hardness; *σ* = strength; *ε_b_* = elongation at break; ↑ = increase; ↓ = decrease; ≈ = no significant change.

## Data Availability

No new data were created or analyzed in this study. Data sharing is not applicable.
